# Neuropeptide and cytokines expression in long COVID-19 related neuropsychological sequelae: insights into NK1R-mediated neuroinflammation and *in silico* therapeutic targeting

**DOI:** 10.3389/fncel.2026.1763029

**Published:** 2026-03-26

**Authors:** Muhammad Abdullah, Anam Naz, Leah R. Reznikov, Javed Anver Qureshi, Ammarah Hasnain, Ayesha Obaid, Amjed Ali

**Affiliations:** 1 Institute of Molecular Biology and Biotechnology, The University of Lahore, Lahore, Pakistan; 2 Department of Physiological Sciences, The University of Florida, Gainesville, FL, United States; 3 Faculty of Biological Sciences, Lahore University of Biological and Applied Sciences, North Tulspura, Lahore, Pakistan; 4 Department of Medical Lab Technology, The University of Haripur, Haripur, Pakistan; 5 University Institute of Physical Therapy, The University of Lahore, Lahore, Pakistan

**Keywords:** cytokines, long COVID-19, neuroinflammation, neuropeptides, neuropsychological sequelae, NK1R antagonist

## Abstract

**Background:**

Long COVID-19 causes neurophysiological, cardiopulmonary, and musculoskeletal issues. Increased neuropeptides and cytokines lead to neuroinflammation, resulting in neurocognitive impairments, fatigue, depression, anxiety, and severe cognitive deficits. The Neurokinin 1 receptor (NK1R) is a cellular receptor for the neuropeptide Substance P, and its dysregulation links to neuropsychological issues despite antipsychotic use.

**Objectives:**

In the present study, neuropsychological sequelae related to long COVID-19 were screened and the expression of related neuropeptides and cytokines was evaluated. Additionally, potential drugs have been evaluated computationally to reduce neuroinflammation in long COVID-19.

**Methods:**

After informed consent, subjects were screened by a medical physician for long COVID-19 in an outdoor patient clinic. Various biological scales were used to assess and categorize the severity of neuropsychological symptoms related to long COVID-19. After that, peripheral blood samples were collected from subjects using ELISA and RT-qPCR. Nine drugs were selected and subjected to virtual screening to identify potential drug antagonists for NK1R. The key drug-like properties, safety profile, pharmacokinetic analysis, and biological activity of the identified hits were assessed.

**Results:**

In this study the mean age of 90 patients (60% males and 40% females), was 33 ± 5 years in the symptomatic group and 31 ± 6 years in the asymptomatic long COVID-19 group for <40 years age-group. Whereas, the mean age of >40 years age-group was 58 ± 10 years in the symptomatic group and 54 ± 11 years in the asymptomatic long COVID-19 group. The minimum persistence of duration of long COVID-19 related symptoms in the <30 weeks group was observed to be 19 ± 6 weeks, while 44 ± 6 weeks in the >30 weeks group of symptomatic long COVID-19. A total of 48% patients had fatigue, 47% complained about headache, 28% had anxiety, 25% faced depression, 20% had psychosocial distress, 20% felt discomfort, and 13% had cognitive impairment. A total of 10% had reported dizziness sequelae among long COVID-19 survivors. Experimental data showed upregulation of IL-6, IL-10, and SP in both symptomatic and asymptomatic individuals compared with controls (*p* < 0.001). Drug screening analyses revealed aprepitant (−9.3 kcal/mol) and N- acetyl- L- tryptophan (−8.7 kcal/mol) stable interactions with NK1R and maintaining molecular dynamics stability (RMSD: 1.5–2.2 Å; RMSF 0.8–1.4 Å; Rg approximately 21.6 Å). These compounds also demonstrated favorable blood-brain barrier permeability and pharmacokinetic profiles, suggesting their potential as therapeutic antagonists for treating prolonged COVID-related neuroinflammation.

**Conclusion:**

IL-6, IL-10, and SP are found to be deregulated in long COVID-19 leading to neurophysiological sequelae. To overcome neuropsychological sequelae, binding of SP to NK1R can be hindered using aprepitant and N-Acetyl-L tryptophan which has been evaluated computationally and may require further *in vivo* and *in vitro* studies for validation.

## 1 Introduction

Long COVID-19 related neuropsychological sequelae have become a prominent concern globally, impacting a significant number of survivors months to years after initial infection (Huff et al., 2025). As of the current date, over 164 million confirmed cases of COVID-19 have been reported, and at least 3.4 million individuals have succumbed to the disease (WHO, 2024). COVID-19 infects all cells of the body, including brain cells and causes neuro-inflammatory symptoms even after a 12-week recovery from the COVID-19 onset (Al-Aly et al., 2024; Ali et al., 2023). Long COVID-19 syndrome refers to symptoms persisting beyond 12 weeks after SARS-CoV-2 infection and cannot be explained by any alternative diagnosis (WHO, 2024). Neuropsychological sequelae are characterized by cognitive impairments such as memory loss, depression, anxiety and emotional and behavioral dysfunction, as well as neuropsychiatric symptoms including anxiety, depression, and fatigue. These persistent symptoms are widely reported in both hospitalized and non-hospitalized individuals (Almeria et al., 2024; Efstathiou et al., 2022; Panagea et al., 2025).

Cognitive deficits of long COVID-19 manifest prominently in domains of executive function, attention, information processing speed and memory, often described under the umbrella of “brain fog” (Koch et al., 2026; Panagea et al., 2025). Longitudinal studies indicate that while some patients experience partial cognitive recovery, a substantial proportion continue to suffer persistent impairments that significantly affect daily functioning (Aretouli et al., 2025). Neuropsychological evaluations reveal heterogeneity in impairment profiles, suggesting multifactorial etiologies influenced by patient age, severity of acute illness and comorbidities (Pettemeridou et al., 2025). Significantly, subjective cognitive complaints sometimes diverge from objective test results, suggesting a contributory role for psychological distress and fatigue. Neuropsychological sequelae are associated with neuroinflammation that persists due to dysregulation of neuropeptide and cytokine expression (Davis et al., 2023). Mechanistically, neuroinflammation, microvascular injury and disruption of the blood-brain barrier are implicated in the cognitive and psychiatric symptoms of long COVID-19 (Efstathiou et al., 2022). Systemic hyperinflammation triggered by SARS-CoV-2 induces sustained neuroimmune activation, leading to neuronal dysfunction (Talkington et al., 2025). Neuroimaging studies confirmed structural and functional abnormalities in brain regions responsible for cognition and mood regulation in affected patients. Additionally, microvascular damage, which impairs cerebral perfusion, is recognized as a crucial factor in exacerbating cognitive decline (Talkington et al., 2025; Zhao et al., 2024).

COVID-19 infections have a tendency to invade the nervous system, leading to significant anoxic brain injury, disrupted metabolic processes and an imbalance between free radicals and antioxidants (Zhang et al., 2022). Studies have found that SARS-CoV-2 infection can cause oxidative stress and neuroinflammation by disrupting iron metabolism, leading to an excess production of reactive oxygen species (Sarkar et al., 2023). This oxidative stress damages neuronal cells, causes cerebral ischemia and disrupts metabolic function (Bowen et al., 2023). A noxious stimulus triggered the release of a neuropeptide at the nerve ending which might have triggered neuroinflammation in the brain and exacerbated neurophysiological sequelae (Henri et al., 2022). Substance P is a neuropeptide, and upon its release, it combines with its cellular receptor neurokinin 1 (NK1R) and is involved in systemic inflammation and causes systemic complications, especially cardio-respiratory, musculoskeletal, and respiratory issues among COVID-19 survivors (Mehboob et al., 2024). The NK1R belongs to the tachykinin receptor family, has a seven-transmembrane domain and is involved in pain transmission, the stress response and inflammation.

Sarkar et al. (2023) emphasized that viral invasion and the cytokine storm impair antioxidant defenses, resulting in substantial oxidative damage to the brain. Persistent elevation of plasma cytokines, especially interleukin-4 (IL-4) and IL-6, has been linked to inflammatory changes (Hunter and Jones, 2015; Mazza et al., 2020). These neuroimmune signaling alterations contribute to a wide range of mental health and neurological issues in long COVID-19 syndrome. Many recovered individuals report high rates of mental health problems, including post-traumatic stress disorder (28%), depression (31%), anxiety (42%), and insomnia (40%). Other neurological symptoms include headaches, fatigue, cognitive impairment ("brain fog"), dizziness, memory deficits, confusion, dysautonomia, and attention difficulties (Sun et al., 2021). Addressing these complex neurological and psychiatric aftereffects of COVID-19 can help healthcare providers improve quality of life and promote functional recovery, ultimately reducing the long-term burden on individuals and healthcare systems. Biomarkers have been critical in elucidating the neurobiological alterations underlying long COVID-19 sequelae. The upregulation of interleukin-10 (IL-10) and reductions in neurotrophic factors, such as nerve growth factor (NGF), are consistently observed in patients with persistent symptoms, indicating ongoing neuroinflammation and impaired neural repair (Moen et al., 2025). NICE guidelines recommend integrating biomarker evaluation with clinical and neuropsychological assessment to enhance diagnostic precision and to enable tailored treatment and rehabilitation plans (Venkatesan, 2021). Furthermore, blood-based biomarkers combined with advanced neuroimaging hold promise for early detection of individuals at risk (WHO, 2025; Guillen et al., 2024). Inflammatory (IL-06, IL-1β, TNFα) and anti-inflammatory cytokines are also produced and contribute to systemic inflammation (Schultheiß et al., 2022). WHO defines Long COVID-19 Condition (PCC) as symptoms lasting at least 2 months, starting within 3 months of acute COVID-19 illness, and requiring systematic monitoring and intervention strategies (WHO, 2025). The National Institute for Health and Care Excellence (NICE) guidelines emphasize a multidisciplinary approach that encompasses physical, cognitive and psychological assessments to manage these complex sequelae effectively (NICE, 2025). In clinical practice, comprehensive neuropsychological rehabilitation programs focused on cognitive training and compensatory strategies and have shown benefit in alleviating symptoms and improving functional outcomes (Lana et al., 2024). Emotional and psychological support addressing anxiety and depression is also paramount (García-Molina et al., 2024).

Neuropsychological findings help understanding structural and functional brain changes, guiding targeted neurorehabilitation to improve outcomes (Català et al., 2024). The goal of this study is to identify neuropsychological effects related to long COVID-19 and their biomarkers. Additionally, to explore and evaluate potential drugs by *in silico* analysis to reduce neuroinflammation in long COVID-19. The present study emphasized the importance of early detection and systematic assessment of neuropsychological sequelae of long COVID for timely intervention (WHO, 2025). Understanding the dysregulation of neuropeptides and cytokines, prevalence and spectrum of neurological symptoms related to long COVID-19 helps clinicians to tailor the care and systematic plans to mitigate neuropsychological effects. Incorporating biomarker analysis reveals neuroinflammatory mechanisms, such as SP and IL-10, offering insights into the pathophysiology of long COVID-19 and enabling personalized treatments. *In silico* drug analysis focusing on therapeutics targeting the NK explores interventions to reduce SP-mediated neuroinflammation, a key factor in cognitive dysfunction.

## 2 Materials and methods

### 2.1 Study design and participants

After approval from the Ethical Committee of the University of Lahore (REC-UOL-90-I-04-2023), the present study was initiated in outpatient medical clinics of public primary care hospitals in Lahore. The primary aim was to identify and evaluate individuals in the convalescent phase of COVID-19 experiencing both physical and psychosocial issues over the past weeks. Inclusion criteria included a prior diagnosis of COVID-19 confirmed by a nasopharyngeal swab, a positive RT-PCR test for COVID-19, and after 2 weeks, absence of symptoms and negative nasopharyngeal tests (tested 24 h apart) and later developing symptoms after 12 weeks of recovery from acute COVID-19 that other diagnoses could not explain. Initially, patients who had received care during the acute phase and agreed to follow-up at medical outpatient clinics were included. Later, patients meeting the inclusion criteria were recruited from territory evaluations. The patients were divided into three groups: the first group included individuals with a confirmed history of positive RT-PCR for COVID-19 who developed neuropsychological sequelae after 12 weeks of recovery; the second group comprised individuals with a positive RT-PCR history for COVID-19 who did not develop neuropsychological sequelae after 12 weeks; and the third group consisted of healthy controls with no history of COVID-19 infection, no clinical manifestations and no positive RT-PCR test. Patients younger than 18 years, with prior neuropsychological issues, psychiatric disorders, or pregnant females were excluded from the study.

### 2.2 Sample size calculation and sampling technique

A sample size of 90 subjects was calculated to provide 80% power, 6% absolute precision, and an expected prevalence of IL-6 in the population of 72%. A purposive sampling technique was used and each group included 30 patients.


$$\begin{array}{l} n=\frac{\;{Z^{2}}_{1-\alpha /2}\;.\;P\left(1-P\right)}{d^{2}} \end{array}$$

### 2.3 Data collection

With informed consent, medical history, demographic data, COVID vaccination type and dose, duration of recovery from an acute long COVID-19 infection and neurological and physical examinations for neuropsychological symptoms at 12 weeks after COVID-19 infection of included subjects were recorded and evaluated. The Yorkshire Rehabilitation Scale (YRS) was used to measure long COVID-19 symptoms (Sivan et al., 2022). The scale was translated from English to Urdu for better understanding (Google Translator). It was rated on a scale from 0 to 11, where 0 indicates no symptoms and 10 indicates severe symptoms such as breathlessness, fatigue, and cognitive impairment. Furthermore, neuropsychological evaluations were conducted by clinical psychologists and assessed using standardized scales.

### 2.4 Measures

Initially, the Fatigue Assessment Scale (FS) and the Brief Fatigue Inventory (BI) were used to assess fatigue presence and severity. In the structured screening process, fatigue was characterized 10 scale items with cut-off score ≥22 indicate the presence of fatigue (Zhang et al., 2015; Shahid et al., 2011). Fatigue severity was classified based on the mean score: < 1 indicating no fatigue; 1 to < 4 indicating mild fatigue; 4 to < 7 indicating moderate fatigue; and a cut-off score ≥1 indicating the presence of fatigue (Shahid et al., 2011). Secondly, the Migraine Disability Assessment Tool was used to evaluate headache impact, with scores ranging from 0 to ≥21 and a cut-off score ≥6 showed severe headaches (Zandifar et al., 2014; Lipton et al., 2001). Third, the coronavirus anxiety scale was utilized to assess anxiety dysfunction, employing a 5-items scale with each item rated 5 points (0–4). The maximum resulting score was 22 and cut-off score ≥9 indicated greater levels of anxiety (Lee, 2020). Fourth, the depression module of the Patient Health Questionnaire (PHQs-9) was employed to assess depressive symptoms with threshold score ≥10 (Kroenke et al., 2001; Ford et al., 2020). Based on the PHQs-9, patients' depressive symptoms were categorized as minimal (1–4), mild (5–9), moderate (10–14), moderately severe (15–19), and severe (20–27). Fifth, the Montreal Cognitive Assessment (MCA) was employed to assess cognitive dysfunction, characterized by a MCA cut-off score ≥26 (Freitas et al., 2013). The Kessler Psychological Distress Scale (K10) was used to assess psychological distress related to long COVID-19, consisting of 10 items. A cut-off score of ≤ 22 indicated significant psychological distress (Kessler et al., 2003; Andrews and Slade, 2001). The Pittsburgh Sleep Quality Index (PSI) was used to evaluate sleep quality and ranging 0–21 with score of 5 serving as the cuff-off threshold for identifying sleep disturbances (Backhaus et al., 2002; Dautzenberg et al., 2020).

### 2.5 Experimental analysis

#### 2.5.1 Cytokine assay by ELISA

A trained phlebotomist collected a peripheral blood sample from each participant to measure cytokines. IL-6, IL-1β, TNFα, IL-10, and SP were quantified using an ELISA kit (BT Lab, China) according to the manufacturer's instructions.

#### 2.5.2 Real-time quantitative PCR

A peripheral blood sample was centrifuged to obtain serum and total RNA was extracted from the serum using Trizol reagents (Invitrogen, CA, USA). cDNA was synthesized with 500 ng of RNA in a 20 μl reaction mixture using the Thermo High Fidelity Kit (Thermo Scientific, Waltham, MA, USA) according to the manufacturer's instructions. For IL-6, IL-1β, TNFα, IL-10, and TAC1, quantitative real-time PCR (RT-qPCR) was performed using Universal SYBR Green Master Mix (Roche, Basel, Switzerland) on a Light-Cycler 96 system (Roche, Basel, Switzerland). For all remaining samples, the same procedure was carried out.

The primer sequences used in this study are provided in Supplementary File 1. A gradient PCR was performed prior RT-qPCR to validate amplified PCR products of target genes through Gel electrophoresis. A suitable band was cut and cleaned by QIAquick PCR Purification Kit (Qiagen, Germany) and sent to Macrogen Inc. (Seoul, South Korea) for Sanger sequencing. The sequence results of each product of related genes are provided in also Supplementary File 1. Obtained sequences were checked for their homology using nucleotide BLAST (http://www.ncbi.nlm.nih.gov/BLAST) to confirm desired gene identity and verify sequence accuracy.

Subsequently, RT-qPCR was performed using GAPDH as the housekeeping gene and target genes expression were normalized to GAPDH. Relative mRNA levels of target genes were calculated using the 2^−Δ*ΔCt*^ method. All assays included positive and negative controls and were performed in duplicates to ensure accuracy.

### 2.6 Data analysis and statistics

Independent variables (gender, age, duration of infection, biomarkers) were expressed as mean ± standard deviation (Aretouli et al., 2025). The distributions of dependent variables such as neuropsychological sequelae were analyzed and their percentages were displayed in graphs. Normality was assessed before statistical testing. Data that followed a normal distribution were analyzed using Student's *t*-test. Non-normally distributed data were evaluated with the Kruskal-Wallis rank test and the Mann-Whitney U-test. Two-way ANOVA was used for comparisons involving more than two groups. All analyses were conducted using GraphPad Prism and SPSS version 26 (SPSS Inc., Chicago, IL, United States). A *p-value* of less than 0.05 was considered statistically significant.

### 2.7 Drug target identification

Potential therapeutic drugs targeting NK1R; the cellular receptor of SP, were screened as mentioned in following steps.

#### 2.7.1 Selection target

The 3D structure of Neurokinin 1 receptor (NK1R, PDB ID: 6HLO), was retrieved from the protein database (www.drugbank.ca). It was determined by X-ray diffraction at 2.96 Å. The selected structure of NK1R with its bound ligand was used and ligand was subsequently removed using AutoDock-Vina. The pH was set to 7.0, hydrogen ions added and the SDF file converted to PDBQT for docking.

#### 2.7.2 Target preparation and active site prediction

AutoDock-Vina software was used to prepare the NK1R protein for computational analysis by first removing all water molecules and detaching ligands from the active sites (Trott and Olson, 2010). This was followed by energy minimization to achieve a more stable conformation of the NK1R structure. To account for electrostatic properties and accurate interactions, the NK1R protein was protonated by adjusting hydrogen atom positions based on the amino acid side chains. The MOE was adapted for active-site identification at NK1R (PDB ID: 6HLO) and the site-finder predicted a binding pocket and its interactions. During active site determination, dummy atoms were placed on the active site residue at the alpha center of the protein sphere (https://www.chemcomp.com/en/Products.html). Selected ligands were also prepared. The active site score helped identify potential therapeutic sites based on hydrogen-bond donors/acceptors and ligand-binding capacity.

#### 2.7.3 Preparation of ligand molecules

To build a library of NK1R antagonists, nine compounds were chosen to test their potential to reverse long COVID-19-related neurological effects. Discovery Studio Visualizer BIOVIA (https://mybiosoftware.com/biovia-discovery-studio-visualizer-4-5-molecular-visualization.html) was used to generate 2D conformations of selected ligands (lanepitant, fosaprepitant, modafinil, indacaterol, alosetron, aprepitant, N-acetyl-L-tryptophan, netupitant, and selegiline) that were retrieved as SMILES (simplified molecular input line entry system) from PubChem databases and saved in MOL format. To achieve a stable configuration, the energy of each ligand was minimized and partial charges were added.

#### 2.7.4 Grid generation for docking

The receptor grid was generated using PyRx with the NK1R binding sites (https://sourceforge.net/projects/pyrx/). After selecting the active site, the van der Waals radii of receptor atoms were scaled to 1, with a cutoff of 0.25. A site-specific grid was created for the selected residue of ligand length ≤ 20 Å.

#### 2.7.5 Ligand-protein interactions

Ligands were subjected to molecular docking using Auto-Dock Vina to identify high-affinity binders (Eberhardt et al., 2021). The binding affinity (kcal/mol) was evaluated based on ligand-protein interactions. Each ligand was docked with NK1R and the interactions were assessed for affinity, stability and specificity utilizing PyMOL (Rosignoli and Paiardini, 2022). The five most promising compounds advanced to subsequent stages of analysis.

#### 2.7.6 Visualization of molecular interactions

Ligand interactions were visualized using PyMOL to examine key interactions, including hydrogen bonds, hydrophobic interactions, and electrostatic forces. Each ligand-receptor complex was analyzed for essential interactions, including hydrogen bonds, hydrophobic contacts, and electrostatic forces.

#### 2.7.7 Molecular dynamics simulations

Molecular dynamics simulations were conducted to assess the interactions of five ligands (Indacaterol, alosetron, netupitant, aprepitant, and N-Acetyl-L tryptophan) with the NK1R protein. Partial atomic charges for ligands were calculated using the Antechamber module in AMBER 20 (Arantes et al., 2021). Subsequently, the LEaP module was employed to add missing hydrogen atoms, neutralize the system, solvate the complexes and generate the required parameter and coordinate files for molecular dynamics simulations. The protein components were modeled with the ff14SB force field, while the ligands were parameterized with the generalized AMBER force field (GAFF). Protonated protein structures were neutralized with suitable counterions (Cl^−^ or Na^+^) and each complex was solvated in an octahedral TIP3P water box with a 10.0 Å buffer. These solvated systems were saved as PDB files and all necessary topology and coordinate files were created. Before running the MD, the minimization involved three steps to fix steric clashes. First, ions and solvated water were optimized, then pocket residues, including backbone amino acids and finally, the whole system was relaxed to relax protein-ligand complexes. Each step used 2,500 steepest descent steps followed by 5,000 conjugate gradient steps. After minimization, the system was heated gradually from 0 to 300 K, then equilibrated at 300 K using Langevin dynamics with a collision frequency of 1 ps^−1^ and a force constant of 10 kcal mol^−1^ Å^−2^. The MD phase was conducted under the NPT ensemble at 300 K and 1 atm for 50 ns. The stability and interactions of docked complexes of prioritized inhibitors with their particular targets were assessed by simulating the actual condition for the protein ligand complex in the presence of solvent, membrane, and ions.

The AMBER software suite (https://www.ambermd.org/) was used to predict the optimal binding poses through molecular docking. Each selected docked complex (drug with NK1R) underwent energy minimization, equilibration and molecular dynamics simulations during the production phase. The resulting trajectories were analyzed for root-mean-square deviation (RMSD), root-mean-square fluctuation (RMSF) and radius of gyration (Rg). These analyses, spanning 50 ns, aimed to evaluate the complex's structural stability and flexibility (Yoo et al., 2025). All MD simulation analyses included a detailed examination of hydrogen bonding throughout the simulation and graphical plots were also generated using the AMBER suite. These analyses enhance understanding of the dynamic stability, conformational flexibility, and binding behavior of each study complex. Visualization was performed using Discovery BIOVIA software (Hollingsworth and Dror, 2018).

#### 2.7.8 Pharmacokinetic profiling (ADMET)

After performing docking and interaction analysis of the top five ligands, the aim was to identify key features responsible for binding affinity and interaction (Yi et al., 2024). The drug-likeness properties of ligands were assessed using Swiss ADME and ProTox-III (Banerjee et al., 2024; Daina et al., 2017). This web platform evaluates compounds based on descriptors like molecular weight, logP, rotatable bonds, hydrogen bond acceptors and donors, rule violations, and TPSA. To determine drug-likeness, various filters were applied, including Lipinski's, Veber's, Egan's, and Muegge's rules. Lipinski's criteria: no more than 10 hydrogen bond acceptors, 5 donors and a molecular weight below 500 Da. Veber's rule: TPSA ≤ 140 Å^2^ and ≤ 10 rotatable bonds. Egan's filter: TPSA ≤ 140 Å^2^ and logP between −1 and 6. Muegge's rule: molecular weight 200–600 Da, logP −2 to 5, 1–15 rotatable bonds, TPSA < 150 Å^2^. ProTox-III was used to evaluate toxicity with reference drug. The top five with the highest binding affinity were selected and subjected to ADMET and molecular dynamics simulations for 50 ns. At last, drugs were selected that passed ADMET analysis and met appropriate RMSD, RMSF, Gry, and hydrogen-bonding criteria.

## 3 Results

A total of 90 patients were included in present study with 60% being male and 40% females. The mean age in the < 40 years group was 33 ± 5 years for the symptomatic group and 31 ± 6 years for the asymptomatic long COVID-19 group. The mean age in the symptomatic group was 58 ± 10 years and 54 ± 11 years in the asymptomatic long COVID-19 group. The minimum duration of symptoms related to long COVID-19 in the < 30 weeks group was 19 ± 6 weeks and 44 ± 6 weeks in the >30 weeks group of symptomatic long COVID-19—see the other details in Table 1.

**Table 1:** Demographic information analysis in long COVID-19 subjects.

**S. No**	**Demographic data**	**Description**
**1**.	**Total Subjects (males and females)**	*n* = 90
	(a)Symptomatic	*n* = 30
	(b)Asymptomatic	*n* = 30
	(c)Controls	*n* = 30
**2**.	**Gender: (males and females)**	
	(a)Symptomatic	(18, 12)
	(b)Asymptomatic	(18, 12)
	(c)Controls	(18, 12)
**3**.	**Age (Years)**	
	(a)Symptomatic	48 ± 16
	(b)Asymptomatic	37 ± 13
	(c)Controls	33 ± 11
**4**.	**Age groups (years) (**<**40**, ≥**40)**	
	(a)Symptomatic	33 ± 05, 58 ± 10
	(b)Asymptomatic	31 ± 06, 54 ± 11
	(c)Controls	25 ± 02, 46 ± 04
**5**.	**Duration (**<**30 &** ≥**30) weeks**	
	(a)Symptomatic	19 ± 06 & 44 ± 06
	(b)Asymptomatic	18 ± 08 & 42 ± 06
**6**.	**COVID-19 vaccination**	**90(100%)**
**7**.	**Number of doses received**	
	1	Nil
	2	67%
	3	33%
**8**.	**Type of vaccine received**	**%**
	Sinovac	62
	Pfizer	06
	Sinopharm	32
**9**.	**Symptoms**	**%**
	Fatigue	48
	Headache	47
	Anxiety	28
	Depression	25
	Psychosocial distress	20
	Discomfort	20
	Cognitive impairment	13
	Dizziness	10

Long COVID-19 sequelae. These symptoms lasted 44 ± 6 weeks. A total of 48% had fatigue, 47% headache, 28% had anxiety, 25% had depression, 20% had psychosocial distress, 20% had discomfort, and 13% had cognitive impairment. A total of 10% had reported dizziness sequelae among long COVID-19 survivors.

### 3.1 Expression analysis

**Figure 1:**
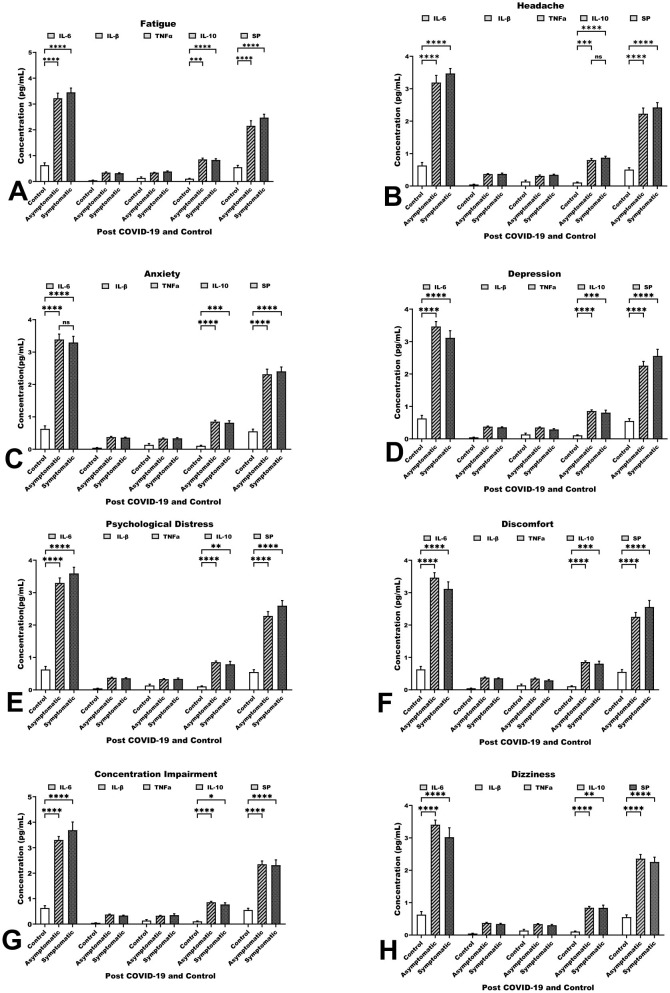
This figure shows ELISA-based concentrations (pg/mL) of IL-6, IL-1β, TNFα, IL-10, and SP across neuropsychological sequelae: **(A)** Fatigue, **(B)** Headache, **(C)** Anxiety, **(D)** Depression, **(E)** Psychological Distress, **(F)** Discomfort, **(G)** Concentration Impairment, and **(H)** Dizziness. Variation in cytokine and neuropeptide protein levels associated with neuropsychological symptoms. *p < 0.05, **p < 0.01, ***p < 0.001, ****p < 0.0001, ns: non-significant.

The ELISA analysis measured biomarker levels (pg/mL) by gender, age and long COVID-19 duration, with no significant differences except for slightly higher levels in males, those over 40 and shorter duration cases as shown in Table 2. The analysis also quantified IL-6, TNFα, IL-1β, IL-10, and SP in long COVID-19 neuropsychological issues, which showed elevated levels in symptomatic individuals compared with asymptomatic individuals and controls (Figure 1). IL-6 and SP were notably elevated across symptoms like headache, anxiety, cognitive, and psychological symptoms (*p* < 0.01). These suggest that IL-6 and SP are linked to complication severity, with IL-6 higher in asymptomatic cases and SP higher in symptomatic cases. The biomarkers IL-6, TNFα, IL-10, and SP may relate to these symptoms while IL-10 was less elevated, indicating a limited anti-inflammatory response. These findings point to ongoing immune activation and sensory signaling pathways in long COVID-19 neuropsychological issues.

**Table 2:** Estimation (pg/mL) of biomarkers by gender, age, and length of long COVID-19.

**Biomarkers**	**Study Groups**	**Gender Mean** ±**SD**		***F***	***p***	**Age Mean** ±**SD**		***F***	***p***	**Duration Mean** ±**SD**		***F***	***p***
		**Male**	**Female**			<**40**	≥**40**			<**30**	≥**30**		
IL-6 (pg/mL)	Symptomatic	3.76 ± 0.64	3.65 ± 0.62	0.07	0.79	3.56 ± 0.55	3.84 ± 0.67	2.94	0.09	3.72 ± 0.70	3.72 ± 0.56	0.37	0.55
	Asymptomatic	2.45 ± 0.52	2.90 ± 0.07			2.36 ± 0.48	3.0 ± 0.17			2.47 ± 0.50	2.78 ± 0.58		
	Control	0.67 ± 0.21	0.49 ± 0.10			0.63 ± 0.15	0.65 ± 0.10			-	-		
IL-1β (pg/mL)	Symptomatic	0.35 ± 0.05	0.42 ± 0.15	0.03	0.95	0.35 ± 0.05	0.39 ± 0.13	0.91	0.31	0.41 ± 0.13	0.34 ± 0.03	2.09	0.16
	Asymptomatic	0.36 ± 0.03	0.36 ± 0.18			0.37 ± 0.03	0.35 ± 0.13			0.37 ± 0.07	0.33 ± 0.01		
	Control	0.16 ± 0.13	0.07 ± 0.04			0.11 ± 0.07	0.19 ± 0.18			-	-		
TNFα (pg/mL)	Symptomatic	0.31 ± 0.1	0.37 ± 0.08	1.67	0.21	0.35 ± 0.09	0.32 ± 0.10	0.02	0.89	0.32 ± 0.10	0.35 ± 0.09	0.03	0.86
	Asymptomatic	0.31 ± 0.1	0.41 ± 0.10			0.33 ± 0.11	0.34 ± 0.15			0.34 ± 0.11	0.29 ± 0.15		
	Control	0.05 ± 0.02	0.03 ± 0.01			0.04 ± 0.01	0.06 ± 0.03			-	-		
IL-10 (pg/mL)	Symptomatic	0.89 ± 0.24	0.88 ± 0.19	0.03	0.85	0.89 ± 0.24	0.89 ± 0.21	0.69	0.41	0.87 ± 0.19	0.91 ± 0.26	2.47	0.13
	Asymptomatic	0.74 ± 0.17	0.76 ± 0.10			0.71 ± 0.16	0.82 ± 0.14			0.70 ± 0.15	0.94 ± 0.04		
	Control	0.12 ± 0.05	0.07 ± 0.04			0.09 ± 0.05	0.13 ± 0.06			-	-		
SP (pg/mL)	Symptomatic	2.70 ± 0.51	1.74 ± 0.45	0.31	0.57	2.70 ± 0.60	2.72 ± 0.39	0.51	0.48	2.75 ± 0.38	2.67 ± 0.60	1.18	0.29
	Asymptomatic	1.49 ± 0.30	1.46 ± 0.11			1.54± 0.29	1.35 ± 0.21			1.54 ± 0.27	1.22 ± 0.13		
	Control	0.61 ± 0.21				0.60 ± 0.27	0.48 ± 0.13			-	-		

SD, Standard Deviation; *F*, ANOVA *F* value; *p*, significance value.

**Figure 2:**
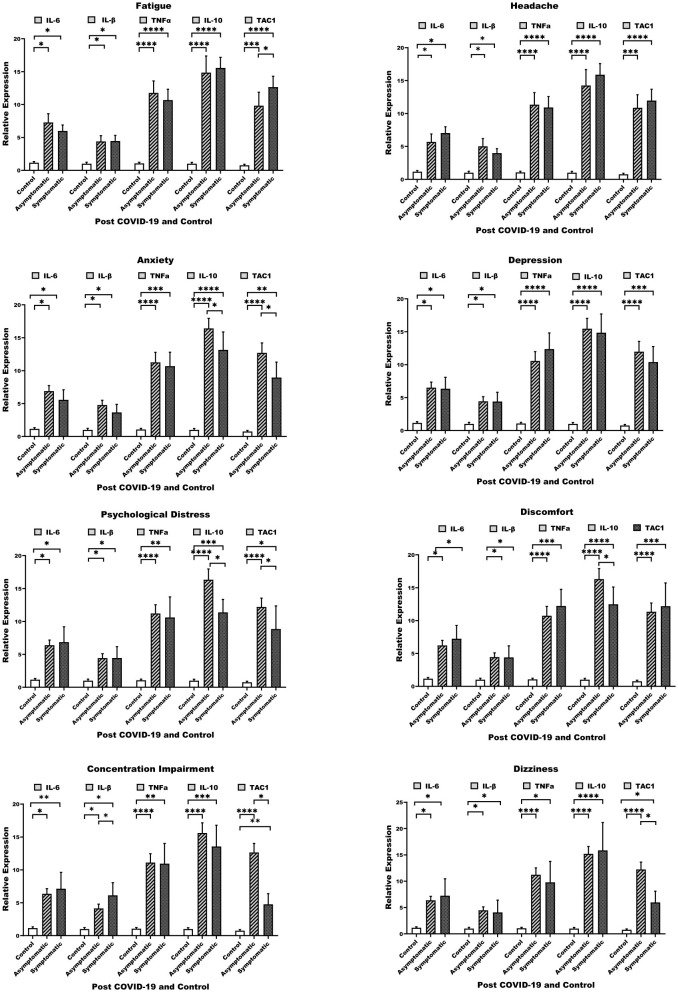
IL-6, IL-1β, TNFα, IL-10, and TAC1 gene expression across various neuropsychological sequelae: **(A)** Fatigue, **(B)** Headache, **(C)** Anxiety, **(D)** Depression, **(E)** Psychological Distress, **(F)** Discomfort, **(G)** Concentration Impairment, and **(H)** Dizziness. The data reflect gene expression patterns associated with neuropsychological symptoms. *p < 0.05, **p < 0.01, ***p < 0.001, ****p < 0.0001.

The rt-qPCR analysis of gene expression in long COVID-19 patients revealed significant increases in IL-6, IL-1β, TNFα, IL-10, and TAC1 levels in both symptomatic and asymptomatic groups compared with controls (Figure 2). In asymptomatic individuals, IL-6 and IL-1β were moderately elevated by 7- and 5-fold, respectively, while TNFα, IL-10 and TAC1 showed more pronounced increases 11, 16, and 13-fold, respectively indicating ongoing inflammatory activity. Symptomatic patients showed persistent elevation of these biomarkers, with IL-6 (6-fold), IL-1β (4-fold), TNFα (11-fold), IL-10 (13-fold), and TAC1 (9-fold), suggesting escalated inflammation and pain pathways. This pattern was consistent across various neuropsychological symptoms such as anxiety, depression, discomfort, cognitive deficits, headaches, and dizziness. The consistent upregulation of these markers indicates ongoing inflammation and neuroinflammation associated with long COVID-19-related neuropsychological issues.

### 3.2 Computational analysis

**Figure 3:**
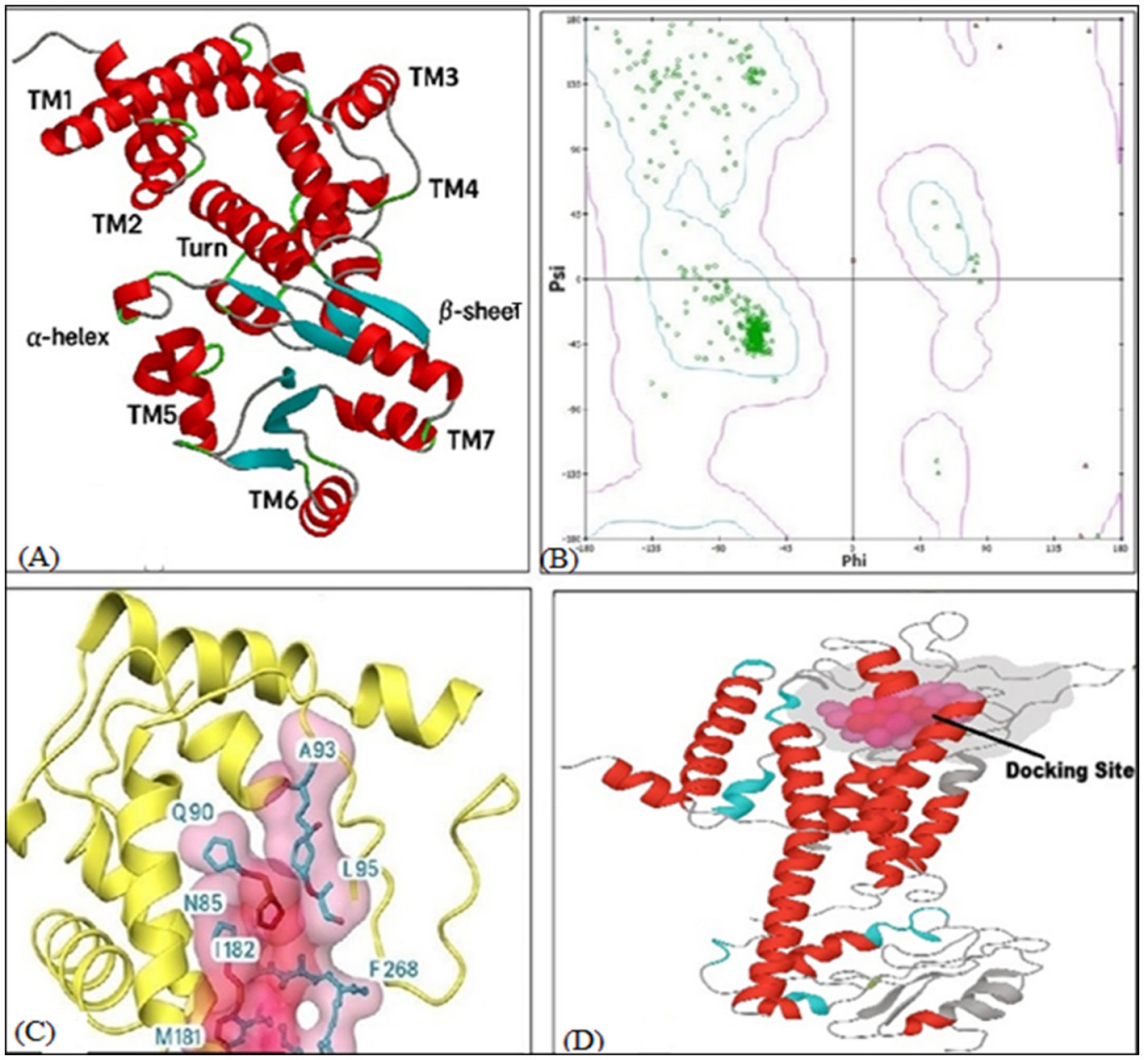
NK1R receptor structure and binding site. **(A)** Red helices represent α-helices common in G-protein coupled receptors like NK1R, Cyan arrows represent β-sheets, green/gray strands are loops or turns non-structured/random coil regions. **(B)** Ramachandran plot for NK1R. This plot shows the distribution of backbone dihedral angles (phi and psi) for residues located within the favored and allowed regions of the NK1R model. **(C)** Binding Sites at NK1R receptor of Substance P (Yellow ribbons/spirals: represent the alpha-helices of the NK1R), a typical G-protein coupled receptor (GPCR, Pink surface). **(D)** It indicates the binding cavity where SP interacts with NK1R, Cyan residues: labeled amino acids forming key interactions with SP (via hydrogen bonding, hydrophobic interactions, or π-π stacking). **(D)** NK1R binding pocket site 1, which represents NK1R's main binding pocket for SP.

This study prepared the NK1R (PDB ID: 6HLO) structure for analysis by modeling missing loops, removing water molecules and ligands and adding hydrogen atoms and charges. After correcting structural defects, optimizing hydrogens, adjusting ionization states and refining bond alignment, the structure's geometry, and stability improved. Energy minimization was done at pH 7. The NK1R protein possesses an extracellular N-terminus and an intracellular C-terminus, characteristic of class A GPCRs. Seven red α-helices represent the GPCR transmembrane domains (TM1–TM7) that traverse the membrane, forming extracellular loops (ECL1–ECL3) on the extracellular side and intracellular loops (ICL1–ICL3) on the intracellular side (Figure 3). The Ramachandran plot indicates most residues are in favored regions, with over 90% suggesting a reliable, properly folded structure (Figure 3B). Few outliers may represent flexible loops or irregularities. The even angle distribution supports the model's robustness, though the exact percentage of residues in favored vs. disallowed regions could give more insight.

### 3.3 Estimation of active sites of NK1R

The druggable sites were predicted and ranked based on-site characteristics and binding affinity scores. Computational Swiss target prediction analysis identified nine key binding residues and five potential ligand-binding pockets which providing insights into their molecular interactions. Using the CB-docking and Fpocket web source platforms, five potential binding pockets with varying volumes and locations were identified (Le Guilloux et al., 2009). The details all binding pockets of NK1R along with their size and coordinates are provided in Table 3.

**Table 3:** Predicted binding pockets of NK1R.

**Pocket**	**Volume (Å3)**	**Center coordinates (x, y, z)**	**Size (Å)**	**Possible role**
C1	1,562	(4.3, −1.0, 29.1)	17 × 19 × 17	The primary binding site for the ligand is given its large volume and central location. Likely accommodates the peptide's bulk.
C2	418	(5.8, 0.4, 62.2)	16 × 14 × 12	Secondary site, possibly for allosteric modulators or auxiliary interactions.
C3-5	343, 263, 167	8.5, −14.0, 68.9 2.1, −25.8, 97.2 −4.6, −5.0, 62.9	12, 15, 13 15, 15, 12 12, 14, 11	Smaller cavities may assist in structural flexibility or signal transduction.

The predictions provide structural insight into the receptor's potential binding regions, guiding further *in silico* docking, mutagenesis studies, or ligand design targeting NK1R. Based on further evaluation, site C1 was selected the best binding pocket and its 3D structure is shown in Figure 3D.

### 3.4 Target selection

**Figure 4:**
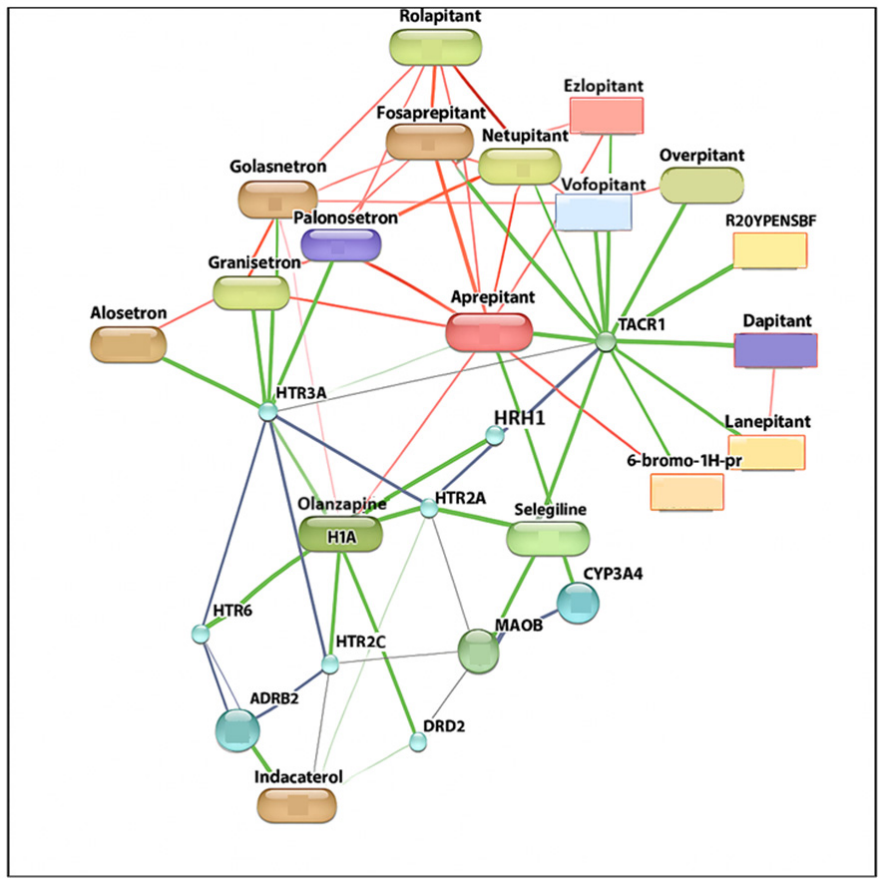
Therapeutic targets interaction. Green lines denote positive interactions, red lines represent negative interactions and blue lines show unknown or predicted associations. The key target TACR1 (NK1R) is highly connected with multiple drugs.

Based on network analysis, NK1R antagonists (FDA-approved) were chosen as the ligands. Details are shown in Figure 4. FDA-approved ligands such as lanepitant, fosaprepitant, modafinil, indacaterol, alosetron, aprepitant, N-acetyl-L-tryptophan (Huff et al.), netupitant, and selegiline were prepared by removing hydrogen atoms and creating potential ionization sites at pH 7 ± 2. The energy of each ligand was minimized and a PDBQT format was designed for molecular docking.

### 3.5 Grid generation

**Figure 5:**
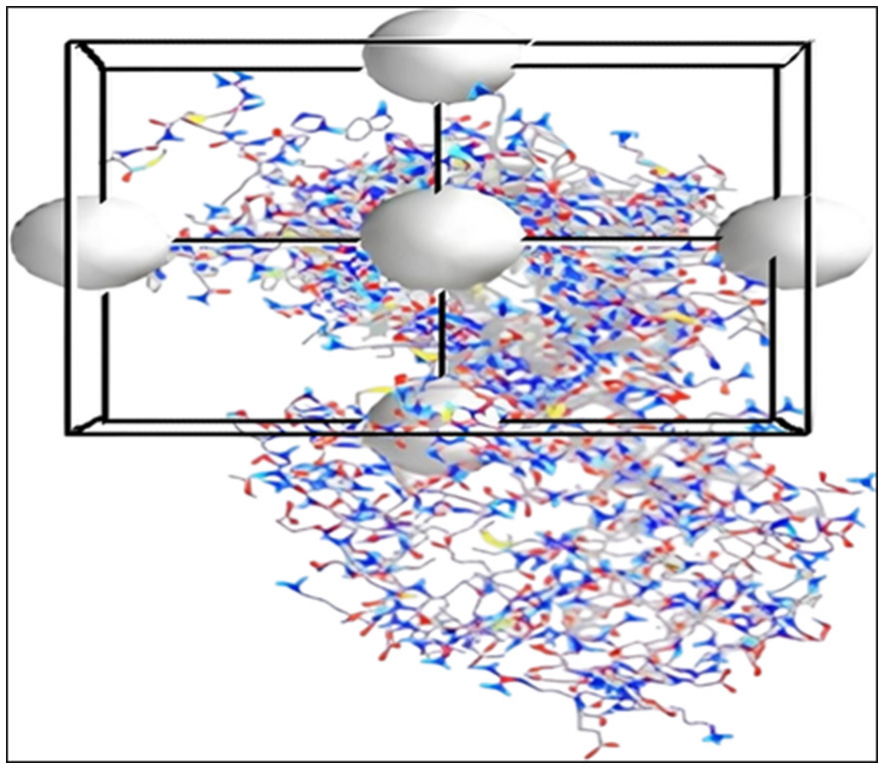
Grid generation for NK1R. The highlighted area with a ball square shows areas of binding with the ligand.

The receptor grid was generated using Discovery Studio and PyRX to select NK1R-binding sites, as shown in Figure 5. To avoid shifting sites, receptor properties, and ligand scores needed an accurate determination. The site was chosen first, with van der Waals radii scaled to 1 and a cut-off of 0.25. A site-centered grid for each ligand minimized errors.

### 3.6 Binding affinity and protein-ligand interaction patterns

**Figure 6:**
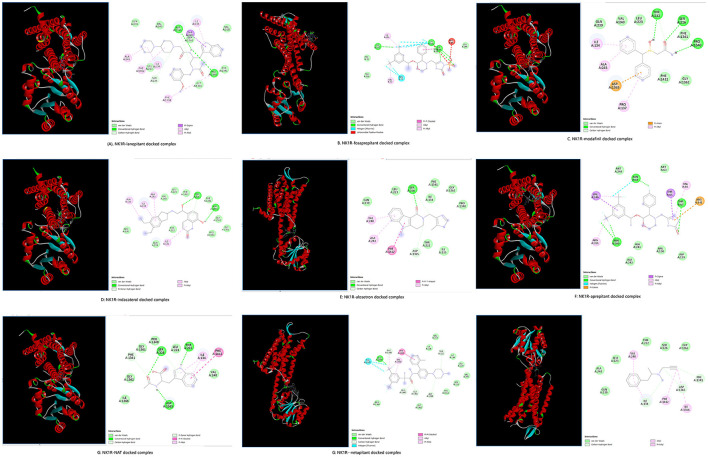
Docked complexes of NK1R with ligands. In each complex, the violet and green color shades indicate hydrogen bonds. Each docked complex shows the 3D **(left)** and 2D **(right)** structure. **(A)** Shows NK1R-lanepitant docked complex, **(B)** displays NK1R-fosaprepitant docked complex. **(C)** Shows NK1R-modafinil docked complex. **(D)** Displays NK1R-indacaterol docked complex. **(E)** Represents the NK1R-alosetron docked complex. **(F)** Shows the NK1R-aprepitant docked complex. **(G)** Displays NK1R-NAT docked complex. **(H)** Represents NK1R-netupitant docked complex. **(I)** Shows NK1R-selegiline docked complex.

The FDA-approved ligands were docked to the A site of NK1R using AutoDock Vina. The study analyzed interactions to evaluate binding affinity, stability, and specificity by examining hydrogen bonds, hydrophobic interactions, and electrostatic forces with PyMOL and UCSF Chimera. The best model was selected based on the highest negative binding affinity score. Ligand structures and 2D interactions are shown in Figure 6. The ligand formed π-π and π-alkyl interactions with phenylalanine, valine, and alanine residues, indicating hydrophobic stabilization. Van der Waals interactions with several residues suggested a well-embedded ligand in NK1R's transmembrane region. 3D visualization confirmed binding within the receptor's domain, involving polar and hydrophobic contacts, which could influence receptor activity.

Overall, interactions suggest that ligands fit well into NK1R, potentially acting as competitive or allosteric inhibitors, mimicking ligand binding. The binding affinity scores for the study drugs (kcal/mol) are presented in Table 4.

**Table 4:** Binding affinity of NK1R with ligands.

**S. No**	**Drug model**	**Binding affinity (kcal/mol)**
1.	6hlo23_Lanepitant_uff_E = 667.52	−7.6
2.	6hlo23_Fosaprepitant_uff_E = 914.30	−7.2
3.	6hlo23_Modafinil_uff_E = 263.47	−6.6
4.	6hlo23_Indacaterol_uff_E = 503.65	−8.3
5.	6hlo23_Alosetron_uff_E = 695.20	−7.9
6.	6hlo23_aprepi_uff_E = 581.10	−6.8
7.	6hlo23_NA-tryptophan_uff_E = 358.39	−6.8
8.	6hlo23_Netupitant_uff_E = 905.11	−7.7
9.	6hlo23_Selegiline_uff_E = 225.43	−5.7

Among the tested ligands, Indacaterol exhibits the highest binding affinity at −8.3 kcal/mol, suggesting it could be a highly potent NK1R ligand. Other strong binders include alosetron (−7.9 kcal/mol), netupitant (−7.7 kcal/mol), and lanepitant (−7.6 kcal/mol), all of which demonstrate favorable interactions with the receptor. Fosaprepitant (−7.2 kcal/mol) also shows moderate binding, while aprepitant and NAT (both −6.8 kcal/mol) and modafinil (−6.6 kcal/mol) exhibit weaker but still notable affinities. In contrast, selegiline (−5.7 kcal/mol) has the lowest binding affinity, indicating poor interaction with NK1R. These computational results highlight that indacaterol, alosetron, netupitant, and aprepitant are the top candidates based on binding strength, with indacaterol being the most promising.

### 3.7 Analysis of ADMET/pharmacokinetics properties

**Figure 7:**
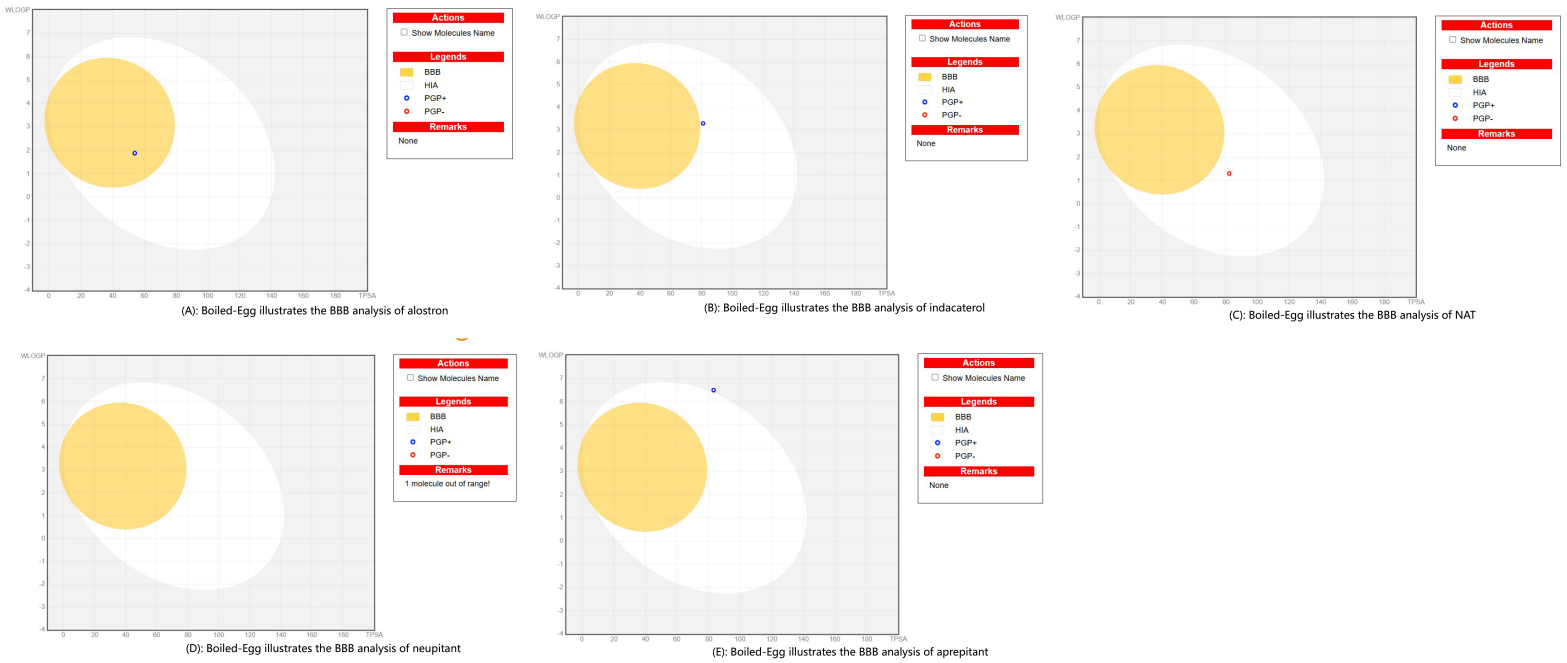
The blood-brain barrier analysis of selected study ligands. **(A)** Boiled-Egg illustrates the BBB analysis of alostron, **(B)** BOILED-Egg displays the BBB analysis of indacaterol, **(C)** BOILED-Egg shows the BBB analysis of NAT-tryptophan. **(D)** BOILED-Egg shows BBB analysis of neupitant, **(E)** BOILED-Egg displays the BBB analysis of aprepitant.

The antagonists of NK1R were screened for their ability to cross the blood-brain barrier (BBB). The BBB permeability and SWISS-ADME (http://www.swissadme.ch/) were evaluated for each drug to assess *in silico* safety biologically. A score of approximately 1 indicates acceptable BBB ability and the results for each drug are shown in Figure 7.

**Figure 8:**
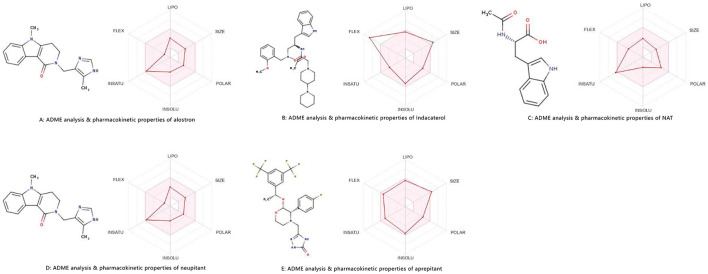
ADME spider plot prediction of docked complexes. **(A)** illustrates ADME analysis & pharmacokinetic properties of alostron. **(B)** Displays ADME analysis for indacaterol & pharmacokinetic properties. **(C)** Shows ADME analysis for NAT & pharmacokinetic properties. **(D)** Shows ADME analysis for neupitant & pharmacokinetic properties. **(E)** Displays ADME analysis for aprepitant & pharmacokinetic properties.

ADME analysis using the “BOILED-Egg” model predicts alosetron's pharmacokinetics. The model estimates gastrointestinal absorption, BBB penetration and PGP substrate status, classifying molecules into yolk (high BBB), egg white (high GI absorption), and outer zone (poor absorption or PGP efflux). Alosetron, a 5-HT3 antagonist for IBS, typically falls in the egg white, indicating high oral absorption but limited BBB access, aligning with its gut-targeted action (Figure 8A). If PGP+ efflux reduces bioavailability, the molecule's hepatic metabolism by CYP2C9 and CYP3A4 complicates the profile. Alosetron's moderate lipophilicity and low polarity support membrane crossing and passive diffusion, overcoming limited solubility. Its small size and conformational flexibility aid tissue penetration, particularly into the enteric nervous system. Nitrogen groups make it a substrate for CYP enzymes, facilitating metabolism to inactive metabolites. Elimination occurs via the renal and biliary routes, supported by its balanced polarity, which influences dosing. Indacaterol, a long-acting beta agonist for respiratory diseases, has a “BOILED-Egg” pharmacokinetic profile (Figure 8B). Its lipophilicity (WLOGP) of around −2 balances hydrophilicity and membrane permeability, like an egg yolk's stability, ensuring solubility, and passive diffusion. The egg white indicates high human intestinal absorption (HIA >80%), aided by its PGP status, which improves bioavailability by reducing efflux. Minimal BBB penetration, like boiling water, with high TPSA (~180 Å^2^), limits CNS side effects and diffusion into non-target tissues. Metabolism and excretion resemble a cracked eggshell, with high TPSA and moderate hydrophilicity favoring renal clearance over hepatic metabolism. Properties such as BBB (none), HIA (high) and PGP indicate indacaterol's optimization for sustained lung action with systemic safety.

The BOILED-Egg plot for NAT shows its ADME properties which highlights its identity and includes legends such as BBB, HIA and PGP+/–, predicting pharmacokinetics. NAT shows moderate intestinal absorption (HIA+) due to balanced lipophilicity (VILOGP 2–4) but probably doesn't cross the BBB due to high polarity. Its high TPSA from polar groups indicates good solubility but limited membrane permeability and its PGP status suggests it isn't a P-glycoprotein substrate, aiding GI absorption. The plot likely places NAT in the high solubility/low permeability quadrant, aligning with its peptide-like nature. No outliers in Figure 8C suggest a predictable ADME profile. Overall, NAT is suitable for systemic delivery, with moderate absorption and limited CNS penetration, making it ideal for peripheral use without crossing the BBB. Its molecular properties reveal a balanced profile from its indole ring, supporting target interaction and stability. Its size favors absorption but insolubility poses formulation challenges. Its metabolism involves amide hydrolysis and indole oxidation with renal excretion. These traits imply good bioavailability via proper delivery with limited CNS effects, making it suitable for peripheral therapies. The molecule's hydrogen-bond donors and acceptors influence its ADME properties, guiding its development as a targeting agent for NK1R.

The BOILED-Egg plot for netupitant an NK1R antagonist used to treat chemotherapy-induced nausea and vomiting, reveals key ADME insights through its parameters (Figure 8D). It shows excellent BBB penetration, supported by optimal lipophilicity balancing permeability and solubility. Human intestinal absorption (Koch et al., 2026) is likely high while P-glycoprotein (PGP) affects its bioavailability and drug interactions. The TPSA suggests moderate polarity, aiding BBB penetration. “1 molecule out of range” may indicate an outlier or property needing formulation attention. VOLOGE is classified as high permeability, which explains its good oral bioavailability and brain penetration, consistent with its clinical use, which involves systemic exposure and CNS activity. ADME profiles guide dosing, accounting for PGP interactions and drug combinations. The visualization confirms drug-like properties and highlights optimization areas for pharmacokinetics. Neupitant, an NK1R antagonist, prevents chemotherapy-induced nausea; its absorption is affected by administration and formulation. Its distribution is vital for crossing the blood-brain barrier to block CNS NK1 receptors. Metabolism by CYP3A4 influences its duration and interactions. Lipophilicity may improve brain penetration but affect solubility flexibility and size impact binding, distribution and clearance. Polarity influences solubility and permeability, while insolubility may require advanced formulation techniques. Stability issues might exist, requiring stabilization during manufacturing or storage. These factors underscore the importance of optimizing neupitant's ADME for effectiveness, safety and patient outcomes.

The BOILED-Egg plot for aprepitant, an NK1R antagonist used to treat nausea, reveals its key ADME features via water/octanol partition coefficient and TPSA values (Figure 8E). With a likely optimal WLOGP (2-4), aprepitant shows balanced lipophilicity for good membrane permeability and solubility. Its TPSA (~80–100 Å^2^) indicates moderate polarity, aiding BBB crossing and systemic absorption. The absence of PGP substrate notation suggests enhanced bioavailability by avoiding PGP-mediated efflux. All parameters fall within expected ranges for CNS-active oral drugs. These properties explain its clinical pharmacokinetics: ~60%−65% oral absorption, tissue distribution (Vd ~70 L) and CNS penetration. Its balanced physicochemical traits influence ADME. Moderate flexibility from its triazoline core allows NK1R binding while maintaining stability. The molecular weight (~534 Da) is near the upper limit for oral bioavailability but remains effective due to optimized lipophilicity. Aromatic bonds enhance receptor interactions and topological polar surface area (~97 Å^2^) aids BBB crossing and solubility. Insolubility (0.02–0.04 mg/mL) presents formulation challenges, addressed via nanosuspensions. Overall, aprepitant exhibits 60%−65% absorption, tissue distribution and metabolism mainly via CYP3A4, exemplifying optimized properties for a neurokinin antagonist.

### 3.8 Toxicity analysis

ProTox-III helps drug design by predicting small molecule toxicity through comparison with reference drugs (Banerjee et al., 2024). It uses parameters like hepatotoxicity, LD50, toxicity class, cytotoxicity, mutagenicity, immunotoxicity, and carcinogenicity to classify toxicity levels. Toxicity is categorized based on LD50: Class II are lethal (5–50 mg/kg), Class III toxic (50–300 mg/kg), Class IV harmful (300–2,000 mg/kg), Class V potentially harmful (2,000–5,000 mg/kg), and Class VI non-toxic (Chibuye et al., 2024). Models are built using *in vivo* and *in vitro* data for hepatotoxicity, carcinogenicity, immunotoxicity, mutagenicity, and cytotoxicity (Ali et al., 2023). In this study, drugs like aprepitant and NAT showed no activity for these toxicities. Indacaterol was mutagenic; alosetron was not tested. Results were compared with reference drugs diclofenac and ibuprofen, which tested positive for hepatotoxicity and immunotoxicity. Toxicity probabilities of compounds along with reference drugs have been shown in Table 5.

**Table 5:** ProTox III - Toxicity analysis of chemicals with reference drugs.

**Compounds**	**Predicted LD50**	**Predicted toxicity class**	**Hepatotoxicity (prediction/probability)**	**Carcinogenicity (prediction/probability)**	**Mutagenicity (prediction/probability)**	**Immunotoxicity (prediction/probability)**	**Cytotoxicity (prediction/probability)**
Indacaterol	369 mg/kg	4	Inactive/0.74	Inactive/0.60	Inactive/0.61	Active/0.80	Inactive/0.66
Alosetron	650 mg/kg	4	Not Calculated	Not Calculated	Not Calculated	Not Calculated	Not Calculated
Aprepitant	1,700 mg/kg	4	Inactive/0.53	Inactive/0.55	Inactive/0.95	Inactive/0.60	Inactive/0.70
NAT	800 mg/kg	4	Inactive/0.66	Inactive/0.78	Inactive/0.99	Inactive/0.88	Inactive/0.73
Netupitant	568 mg/kg	4	Inactive/0.66	Inactive/0.71	Inactive/0.78	Inactive/0.73	Inactive/0.64
Ibuprofen	299 mg/kg	3	Inactive/0.66	Inactive/0.74	Inactive/0.99	Inactive/0.99	Inactive/0.85
Diclofenac	53 mg/kg	4	Active/0.81	Inactive/0.64	Inactive/0.99	Inactive/0.78	Inactive/0.74

### 3.9 Molecular dynamic mapping

**Figure 9:**
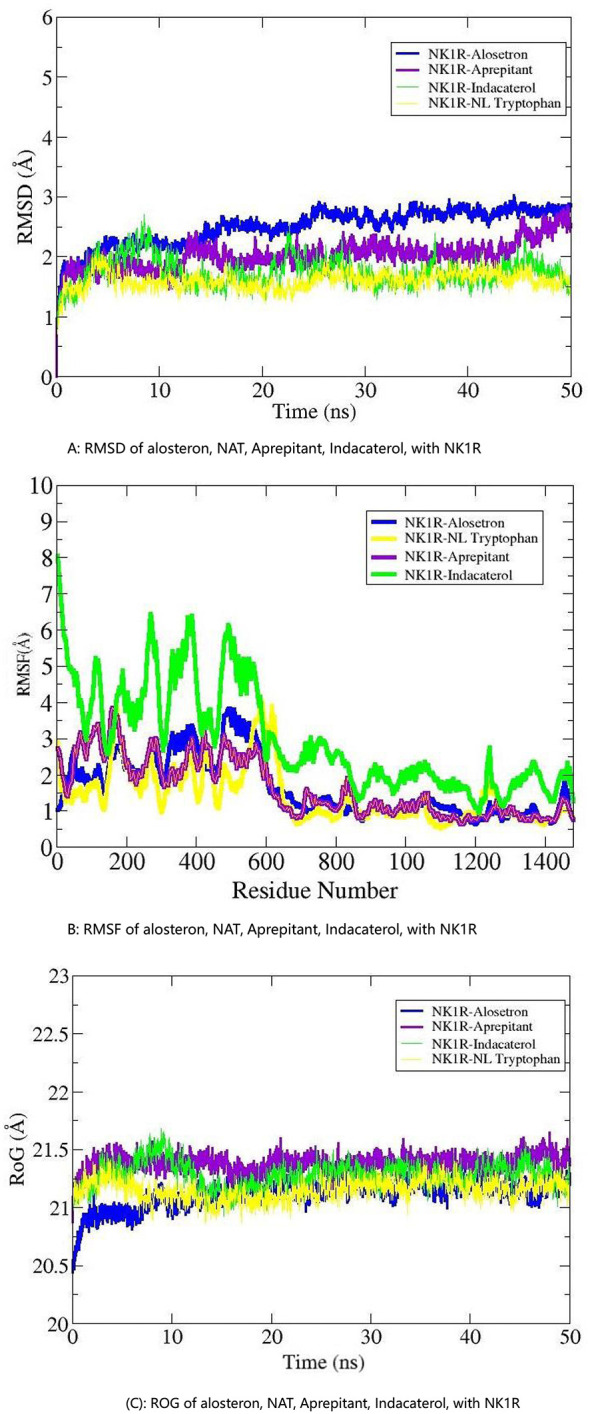
Molecular dynamics simulation. The MD simulation for alosteron, NAT, Aprepitant, Indacaterol, with NK1R, up to 50 ns. **(A)** Showed RMSD, **(B)** showed RMSF, and **(C)** showed Radius of Gyration (RoG Å). Blue represents the alosteron-NK1R, while yellow represents NAT, purple represents aprepitant and green represents the indacatrol-NK1R docked complex.

Structural and dynamic properties of the NK1R–ligand complexes were evaluated from MD simulation trajectories generated by AMBER22 over 50 ns, as shown in Figure 9.

### 3.10 Root mean square deviation (RMSD)

Root mean square deviation (RMSD) analysis assessed conformational changes in NK1R-ligand complexes relative to initial structures. A plateau in RMSD indicates structural stabilization. The NK1R–alosetron complex showed moderate fluctuations, stabilizing at 2–3 Å after ~10 ns, implying stable binding without significant structural change. Conversely, the NK1R–indacaterol complex showed RMSD fluctuations of 3–5 Å, indicating high flexibility or instability, possibly due to suboptimal or transient interactions. The NK1R–NAT complex also had high RMSD (~4–5 Å), suggesting poor stability and weak binding. The NK1R–aprepitant complex started with an RMSD of ~4 Å, which decreased to 2–2.5 Å after 20 ns, indicating initial adjustment before stabilization.

### 3.11 Root mean square fluctuation (RMSF)

RMSF analysis evaluated the structural stability, atomic mobility, and residue flexibility of NK1R-ligand complexes. Peaks in RMSF plots show regions with increased movement, indicating flexibility during simulation. The NK1R–alosetron complex had moderate peaks around residues 200–400 and 800–1,000, likely extracellular loops or near the binding site. Overall RMSF was lower than in the NK1R–NAT complex, indicating more stable binding. The NK1R–indacaterol complex had peaks at residues 200–400 and 1,000–1,200, suggesting increased flexibility near the binding site, possibly due to partial agonist activity or allosteric effects. The NK1R–NL tryptophan complex showed the highest RMSF values, peaking at 9 Å between residues 600–800, implying structural disruption or weak binding. The NK1R–aprepitant complex had low RMSF ( ≤ 5 Å), with minor peaks at residues 400 and 1,200, indicating a rigid interaction, consistent with aprepitant's high-affinity antagonism, which restricts receptor flexibility.

### 3.12 Radius of gyration (Rg)

In this MD analysis, the radius of gyration (Rg) was examined to evaluate the compactness and structural stability of the NK1R-ligand complexes throughout the simulations. Generally, a lower Rg indicates a more rigid structure, while a higher Rg suggests greater flexibility or less compactness. The Rg profile for the NK1R–alosetron complex fluctuated between 20 and 22 Å, indicating a moderately stable interaction with occasional conformational loosening, possibly due to dynamic movements within the binding pocket. The NK1R–indacaterol complex showed the broadest Rg fluctuations (20.5–23 Å), reflecting the least stability and suggesting weak or transient binding. The NK1R–NAT complex started with an Rg of about 22.5 Å, then sharply declined to 0 Å after around 40 ns, indicating ligand dissociation. In comparison, the NK1R–aprepitant complex maintained an Rg around 21 Å throughout, representing the most stable and tightly bound interaction, likely stabilized by strong hydrophobic and van der Waals forces within the receptor pocket.

Table 6 summarizes the molecular dynamics parameters of four ligands bound to the target NK1R. NAT and aprepitant demonstrate high stability, characterized by low RMSD and RMSF values, whereas indacaterol exhibits the highest flexibility and the lowest stability. NAT indicates strong native compatibility and stability, while its high affinity and clinical approval distinguish aprepitant.

**Table 6:** Molecular dynamics parameters of ligand–target complexes.

**Property**	**Alosetron**	**Indacaterol**	**NAT**	**Aprepitant**
Avg. RMSD (Å)	2.8 ± 0.3	1.9 ± 0.2	1.6 ± 0.1	2.0 ± 0.2
Avg. RoG (Å)	20.9 ± 0.2	21.2 ± 0.2	21.1 ± 0.2	21.4 ± 0.2
Max RMSF (Å)	4.2	8.5	3.2	2.4
Stability index	Moderate	Low	High	High

Analysis of MD simulations by using AMBER software demonstrated that aprepitant and NAT form the most stable complexes with NK1R, suggesting their potential as NK1R antagonists. Conversely, indacaterol and alosetron exhibit suboptimal or unstable ligand-binding dynamics.

## 5 Discussion

IL-6, IL-10, TNFα, and SP are found to be associated with systemic complications of long COVID-19. The results predicted SP to be a vital mediator in COVID-19-related inflammation, especially in the respiratory tract and orofacial region, potentially contributing to neurogenic long COVID-19 symptoms. Prior studies have also highlighted the importance of SP, and its link to inflammation and mental health issues such as anxiety and depression (Mehboob et al., 2021). Increased levels of IL-4 and IL-6 have also been reported to cause neurological impairments up to 3 months post-infection (Sun et al., 2021). We have compared neuropsychological symptom scores with biomarker expression in present study and found IL-6, TNFα, IL-10, and TAC1 correlating significantly with gastrointestinal symptoms. Specifically, TNFα, IL-10, and TAC1 mRNA levels were associated with abdominal pain, diarrhea and eating disorders (*p* < 0.001). Understanding pathways involving IL-10 and SP could guide targeted therapies to manage long-term complications. The findings of this study align with those previous studies as well indicating IL-6 with a dual role in the central nervous system, exhibiting both neuroprotective and neurotoxic effects (Kummer et al., 2021). A positive correlation between IL-6 levels and the severity of COVID-19 symptoms and post-acute sequelae have also been reported (Peluso et al., 2021). TNFα was also found associated with long COVID-19 symptoms in this study and it has already been reported to involve in brain connectivity and cognitive decline (Nuber-Champier et al., 2023). Elevated TNFα levels were associated with hippocampal and temporal pole connectivity, affecting cognitive function and contributing to prolonged impairments (Nuber-Champier et al., 2025). IL-10, an anti-inflammatory cytokine, is expressed in long COVID-19 cases. It binds to heterotetrameric receptors (IL-10RA and IL10-RB), activating JAK1 and STAT2 pathways that inhibit T-cell activation. It has already been identified as a key immunological biomarker for COVID-19 severity in various studies and involve in resolving inflammation (Zhou et al., 2020; Han et al., 2020; Queiroz et al., 2024). Elevated levels of IL-6, IL-1β, TNFα, IFN-γ, IL-10, interleukin 2, soluble interleukin 2 receptors, C-reactive protein, MCP-1, serum amyloid A, brain-derived neurotrophic factor and tryptophan, are also found to be linked with depression after COVID-19 (Lorkiewicz and Waszkiewicz, 2021). IL-10 plays a role in resolving inflammation and supporting repair, underscoring its potential as a biomarker for long COVID-19. Common long COVID-19 symptoms include body aches, weakness, joint pain, muscular discomfort and chest pain, and cytokine such as IL-10 could significantly reduce pain and improve quality of life (Bussmann et al., 2022). Whereas, IL-6 trans-signaling has been linked to autoimmune conditions and sleep regulation (Hunt et al., 2021). The connection between IL-6 and sensory deficits, such as smell and taste disorders, is well established and it has been observed that COVID-19 patients recovered smell and taste with lower IL-6 levels, suggesting that reducing IL-6-mediated inflammation may help restore sensory function (Cazzolla et al., 2020). IL-6 also influences gustatory and olfactory pathways at both peripheral and central levels, especially in the thalamus (Vigliar et al., 2022). Elevated IL-6 impairs lymphocyte function, contributing to immune suppression, particularly in severe cases leading to cardiovascular disease, type 2 diabetes and COVID-19 hospitalization. According to Mendelian Randomization studies, IL-6 is dysregulated cardiovascular disease, type 2 diabetes and COVID-19 hospitalization (Bovijn et al., 2020; Xie et al., 2024). The role of SP in COVID-19′s inflammatory response is notably worthy, especially in the respiratory and orofacial regions and its potential role in symptoms and complications (Schirinzi et al., 2023; Esposito et al., 2023). Our findings also verify SP's significant association with long COVID-19 sequelae (*p* < 0.05).

During acute COVID-19, a cytokine storm occurs in symptomatic patients, whereas asymptomatic individuals are less affected due to a more effective immune response. Pro-inflammatory cytokines, including IL-6, IFN-γ, IL-1β, IL-18, and IL-7, as well as chemokines such as IP-10 and MCP-1, are less dysregulated in asymptomatic COVID-19 patients compared with symptomatic patients, but are more than in healthy individuals (Chan et al., 2021). IL-6, IL-1β, and TNFα were less upregulated in asymptomatic individuals, while cytokines such as interferon-γ (IFN-γ) and IL-12 were reduced, indicating a shift toward innate pro-inflammatory signaling without full Th1-type antiviral activation (Tripathy et al., 2021). The level of T cells associated with cytokine production which caused by high CD4+ and CD8+ T cell response in asymptomatic infection to prevent tissue damage and injuries (Le Bert et al., 2021; Peluso et al., 2021). Immune response exhaustion and virus-mediated indirect signaling, including pathogen-associated molecular patterns (PAMPs), cause peripheral dysregulation of neuropeptides and pro-inflammatory cytokines, leading to impaired brain function (Moen et al., 2025). During COVID-19 infection, stimulation of the trigeminal nerve activates microglial responses and also impairs astrocyte function, leading to dysregulated inflammatory cytokine and neuropeptide signaling, which ultimately causes neuroinflammation and neurophysiological sequelae (Slama Schwok and Henri, 2024). The present study data support that IL-6, IL-1β, TNFα, and SP were less dysregulated in asymptomatic long COVID-19 individuals compared with controls. In asymptomatic individuals, increased IL-10 production helps the immune system clear residual viral components and suppresses excessive pro-inflammatory cytokine production (Carlini et al., 2023). The present study also supports that IL-10 production is maintained in asymptomatic long COVID-19 individuals, which helps prevent the dysregulation of pro-inflammatory cytokines and systemic inflammation associated with neurophysiological sequelae. These findings suggest that asymptomatic long COVID-19 is not immunologically inert but is instead characterized by moderate elevations of IL-6, IL-1β, and TNFα alongside regulatory IL-10 and tightly controlled SP mediated neuroimmune signaling. This reflects an optimal host response in which innate immunity contains viral replication and appropriately engages adaptive immune pathways without triggering excessive inflammation or neurogenic symptoms, thereby preserving clinical wellbeing despite the ongoing long COVID-19 condition.

The antagonist potential of indacaterol, alosetron, aprepitant, NAT, and netupitant against the neurokinin-1 receptor (NK1R) have also been evaluated in this study. The results indicate aprepitant and NAT as promising antagonists of NK1R, with favorable binding affinity, dynamic stability, and pharmacokinetic properties. Aprepitant, a clinically established FDA-approved NK1R antagonist, demonstrated firm interaction profiles and previous research highlighted its effectiveness in blocking SP-mediated signaling pathways implicated in nausea, inflammation and pain (Ibrahim et al., 2024; Yoo et al., 2025). The molecular dynamics simulations confirmed the stability of Aprepitant within the NK1R binding pocket and supporting its antagonistic mechanism. NAT has been demonstrated to possess neuroprotective and anti-inflammatory properties, partly through NK1R antagonism, although it is less characterized than aprepitant. Docking and computational molecular dynamics studies confirmed the ability of NAT to bind to NK1R with stability and suggest its potential utility in therapeutic applications targeting neuroinflammation (Thornton et al., 2014). ADMET profiling for both compounds predicted good absorption, blood-brain barrier permeability and low toxicity, underscoring their drug-like characteristics and safety profiles, which are critical factors for potential drug candidates in clinical development (Satarker et al., 2022). In contrast, other compounds, such as indacaterol, alosetron, and netupitant, did not exhibit comparable binding affinities or dynamic stability profiles to those of NK1R antagonists. Indacaterol primarily acts as a beta2-adrenergic agonist, alosetron as a serotonin receptor antagonist, while netupitant, though an NK1R antagonist, was less effective in this comparative study. These findings align with their distinct pharmacological targets and clinical indications, reinforcing the specificity and superior antagonistic potential of aprepitant and NAT at NK1R (Chen et al., 2019; El-Deeb et al., 2023; Rudd et al., 2016). Thus, the integrated computational approach applied for docking, molecular dynamics and ADMET screening substantiates the selection of aprepitant and N-acetyl tryptophan as appropriate NK1R antagonists.

In the context of emerging therapeutics for long COVID-19, the present study findings suggest that computational modulation of the SP–NK1R axis, via drugs such as aprepitant and NAT, may offer distinct advantages over more broadly acting as anti-inflammatory. The primary advantage of aprepitant and NAT is ability of blood–brain barrier permeability and effect on central nervous system to block binding of SP and reduce systemic complication. Antivirals such as remdesivir, molnupiravir, nirmatrelvir/ritonavir (paxlovid), and ensitrelvir primarily lower Long COVID risk by reducing acute phase viral load and organ damage, yet they have limited evidence for treating established long COVID and do not specifically address neuronal stress or neuro inflammation (Krumholz et al., 2025; Neumann et al., 2020). Similarly, JAK inhibitors such as baricitinib and IL 6 blockers (tocilizumab, sarilumab) dampen cytokine signaling and improve outcomes in severe acute COVID-19, thereby indirectly reducing Long COVID burden, but their chronic use safety and long COVID-19 specific efficacy remain uncertain (Preiss et al., 2025; He et al., 2024; Bonilla et al., 2023; Congdon et al., 2023). Metformin activates AMPK pathway, enhance antiviral like effects, and metabolic stabilization. It stands out among long COVID interventions for its robust clinical data showing a 40%−60% reduction in long COVID incidence when administered early in the course of COVID-19 infection (Livieratos et al., 2024). Other modalities, including pentoxifylline, montelukast, pirfenidone, and pirfenidone–nintedanib combinations, focus on microvascular, airway, or pulmonary fibrosis components of long COVID, while bamlanivimab/etesevimab, mRNA vaccines, and IL6 or JAK inhibitors mainly prevent severe acute disease or modulate systemic immunity but remained unusual in long COVID-19 (Català et al., 2024; Bonilla et al., 2023). Naltrexone and low dose aripiprazole used to treat long COVID-19 but still under longitudinal clinical trial (O'Kelly et al., 2022; Naik et al., 2024; Banerjee et al., 2023). A phase II clinical trial in USA revealed that Nirmatrelvir-ritonavir not beneficial in long COVID-19 when compared with placebo-ritonavir (Sawano et al., 2025). Hyperbaric Oxygen therapy (100% oxygen) was used to resolve fatigue related to long COVID-19 and a significant improvement observed, but not suitable due to potential risk of fire hazards and oxygen free radical induced toxicity (Robbins et al., 2021). Aprepitant is a FDA-approved NK1R antagonist with a well characterized safety profile, readily crosses the blood–brain barrier and targets SP-NK1R mediated neuroinflammation and central fatigue pathways implicated in long COVID-19 neuropsychological sequelae. Aprepitant produced rapid symptomatic improvement in fatigue and cognitive complaints in case study on post-acute COVID-19 syndrome which suggests that NK1R blockade may modulate neuroimmune crosstalk in long COVID-19 without broad immunosuppression (Reinoso-Arija et al., 2021). NAT exerts neuro protective and anti-inflammatory effects by inhibiting mitochondrial cytochrome c release and caspase activation, thereby attenuating neuronal apoptosis and oxidative stress. Preclinical studies in ALS and Alzheimer like models show that NAT reduces neuro inflammation, improves cognitive performance, and preserves neuronal integrity which suggests that it may counteract the neuronal stress and synaptic dysfunction observed in long COVID-19 (Sirianni et al., 2015). This neuroprotective and normalizing effect of NAT represents a biologically plausible adjunctive therapy for patients with long COVID-19 who exhibit prominent neuropsychiatric and neuro degenerative symptoms, particularly given its favorable preclinical safety profile and low toxicity. NAT, though less studied clinically, similarly targets the SP-NK1R pathway and may confer additional antioxidant benefits BBB and tolerability profile due to its derivation from an endogenous amino acid. NAT, as a tryptophan derivative, supports neuronal rescue in neuro degenerative models and reducing neuro inflammation and cerebral edema in models of traumatic brain injury (Donkin et al., 2011; Corrigan et al., 2021). Another study revealed that administration of NAT in Alzheimer's disease improved cognitive decline and neuro inflammatory pathways (Satarker et al., 2024). Neurological and musculoskeletal rehabilitation are helpful to overcome post COVID-19 sequelae among survivors. Vanichkachorn et al. (2023) introduced a multi-step rehabilitation strategy at the Mayo Clinic for the COVID-19 rehabilitation program and initially involved psychological support with medical evaluations to address post COVID-19 conditions. They recommended rehabilitation strategies tailored to patients' needs, particularly to address post-exertional malaise and fatigue (Vanichkachorn et al., 2023). However, benefits for long COVID symptoms are less well established, and it does not specifically target neuro inflammatory or neuro degenerative like mechanisms. Therefore, our study suggests aprepitant and NAT may more directly engage neuroinflammatory and neuronal stress pathways that persist beyond the acute phase, offering a complementary strategy to these systemic immunomodulators to mitigate neuropsychological sequelae related to long COVID-19 but both require longitudinal studies.

## 6 Conclusion

COVID-19 impacts multiple organ systems including the brain, leading to persistent neuropsychological symptoms such as headache, depression, anxiety, cognitive impairment, memory issues, and sleep disturbances that last beyond 12 weeks after COVID-19 onset. These long COVID-19 neuropsychological symptoms are associated with ongoing neuroinflammation caused by dysregulation of neuropeptides and cytokines. This study investigated the expression of SP, IL-6, IL-1β, TNFα, and IL-10 in individuals with long COVID-19 and neuropsychological symptoms, asymptomatic survivors and healthy controls. Symptomatic individuals showed significantly higher expression of TAC1, IL-6, TNFα, and IL-10 (*p* < 0.001), whereas IL-1β levels did not differ across groups. Substance P binds to NK1R, part of the tachykinin family, which plays a role in stress, pain, systemic inflammation, and neuropsychological symptoms in long COVID. To explore potential therapies, an *in silico* approach identified pocket 1 as the primary binding site for NK1R through molecular docking and dynamics simulations. Among nine FDA- approved ligands, aprepitant (−9.3 kcal/mol) and NAT (−8.7 kcal/mol) showed the most stable interactions, maintaining molecular dynamics stability (RMSD: 1.5–2.2 Å; RMSF 0.8–1.4 Å; Rg = approximately 21.6 Å). These compounds also demonstrated favorable blood-brain barrier permeability and pharmacokinetic profiles. Computational analysis indicates that aprepitant and NAT have stable docking, passed ADMET and dynamic behaviors with NK1R, suggesting their potential as therapeutic antagonists for treating long COVID-related neuropsychological sequelae. Overall, these molecular and computational findings emphasize the cytokine–neuropeptide axis as a key driver of neuropsychological symptoms and support NK1R antagonism as a promising treatment approach for further *in vitro* and *in vivo* testing.

## Data Availability

The original contributions presented in the study are included in the article/Supplementary material, further inquiries can be directed to the corresponding author/s.
