# Supplementary material for: Neuropeptide and cytokines expression in long COVID-19 related neuropsychological sequelae: insights into NK1R-mediated neuroinflammation and in silico therapeutic targeting
Source: Front Cell Neurosci. 2026 Mar 26;20:1763029. doi: 10.3389/fncel.2026.1763029 (PMC13061724; doi:10.3389/fncel.2026.1763029)

**Supplementary 1**

The Sequences of Primers used in the study

| **Genes** | **Primers pairs** | **Product**  **Size** |
| --- | --- | --- |
| IL-6 | **Forward:** AAAGTATGAGCGTTAGGACA **Reverse:** ATGATCTGGCTCTGAAACAA | 196bp |
| IL-1β | **Forward:** TGGCATTGATCTGGTTCATC  **Reverse:** GTTTAGGAATCTTCCCACTT | 305bp |
| IL-10 | **Forward:** GAGTCCTTGCTGGAGGACTTTA **Reverse:** TGATGTCTGGGTCTTGGTTCTC | 115bp |
| TNFα | **Forward:** TTTCCAGACTTCCTTGAGAC **Reverse:**  TTTCACGGAAAACATGTCTG | 197bp |
| TAC1 | Forward:: CTCGGAGGAACCAGAGAAACT  Reverse: ACAGCTGAGTGGAGACAAGAA | 109bp |

The NCBI-Blast result of chromatograms for target genes is shown in Figure 4.19.


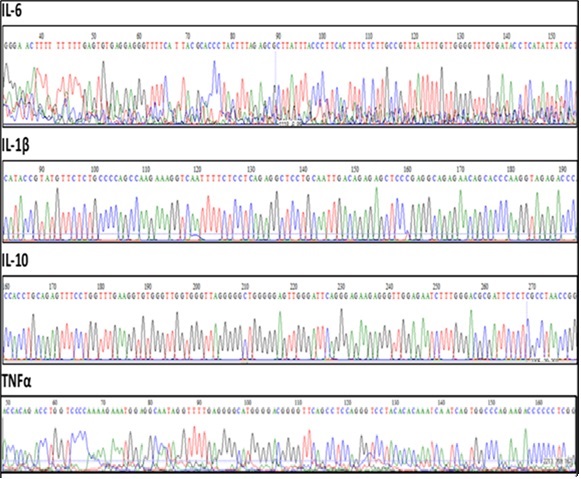


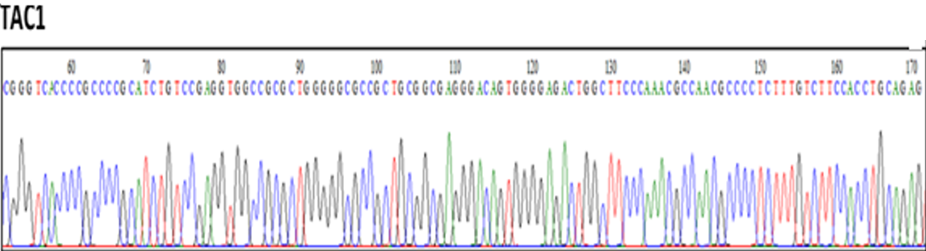


Figure 4.19: Chromatogram of sequenced target genes (IL-6, IL-1β, IL-10, TNFα.

The alignment results of target genes with the reference after BLAST in NCBI are as follows.

**IL-6**

**
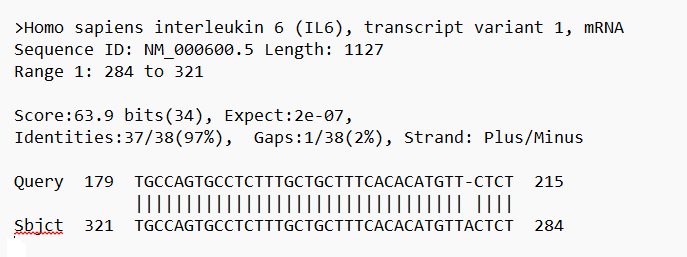
**

**IL-β**


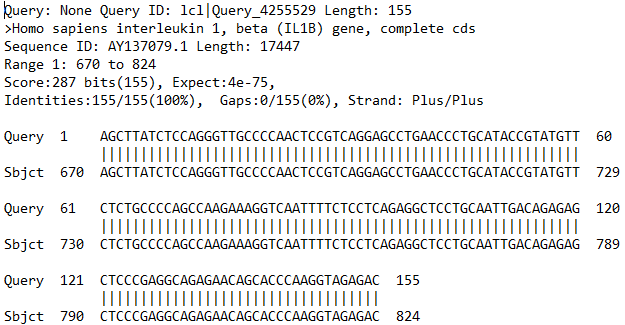


**TNFα**

**
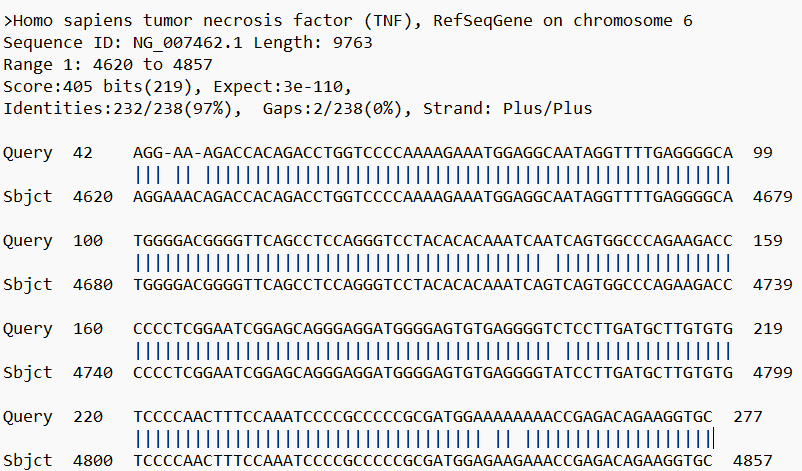
**

**IL-10**


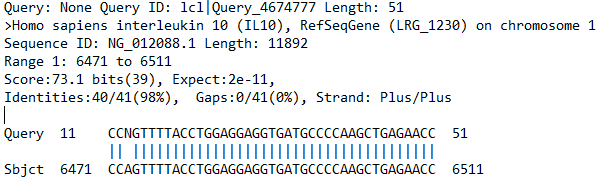


**TAC1**


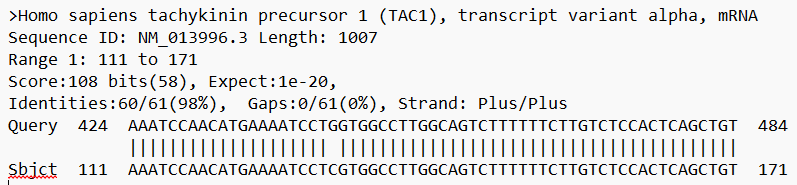

Supplement: Supplementary file 1 [file Data_Sheet_1.DOCX]
