# Supplementary material for: Neuropeptide and cytokines expression in long COVID-19 related neuropsychological sequelae: insights into NK1R-mediated neuroinflammation and in silico therapeutic targeting
Source: Front Cell Neurosci. 2026 Mar 26;20:1763029. doi: 10.3389/fncel.2026.1763029 (PMC13061724; doi:10.3389/fncel.2026.1763029)
Supplement: Supplementary file 2 [file Data_Sheet_2.DOCX]

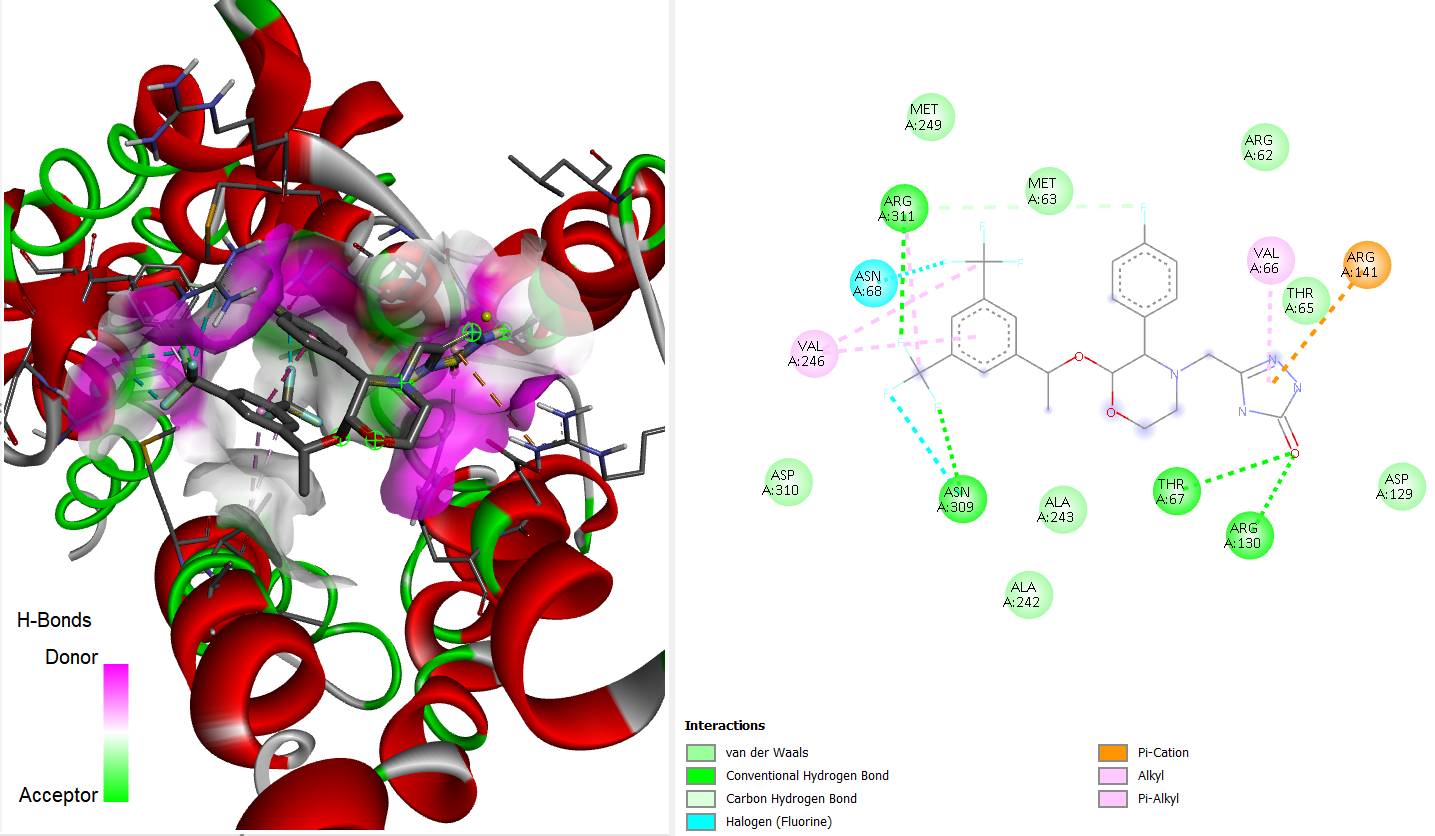


**Figure A.** NK1R-Aprepitant Docked complex 3D and 2D diagram.: Docked complexes of NK1R with ligands. In each complex, the violet and green color shades indicate hydrogen bonds. Each docked complex shows the 3D (left) and 2D (right) structure.


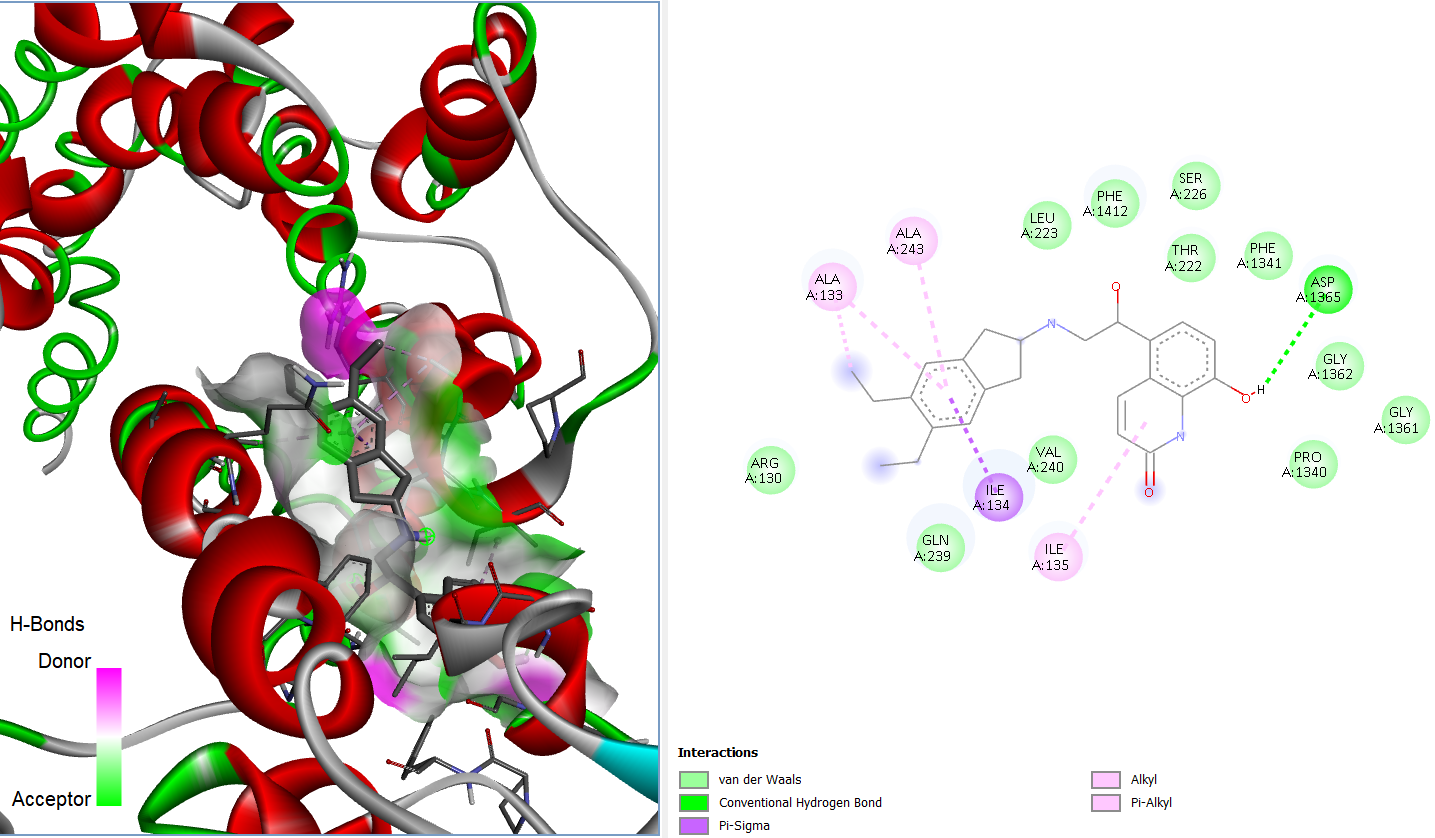


**Figure B.** NK1R-indacaterol Docked complex 3D and 2D diagram.: Docked complexes of NK1R with ligands. In each complex, the violet and green color shades indicate hydrogen bonds. Each docked complex shows the 3D (left) and 2D (right) structure.


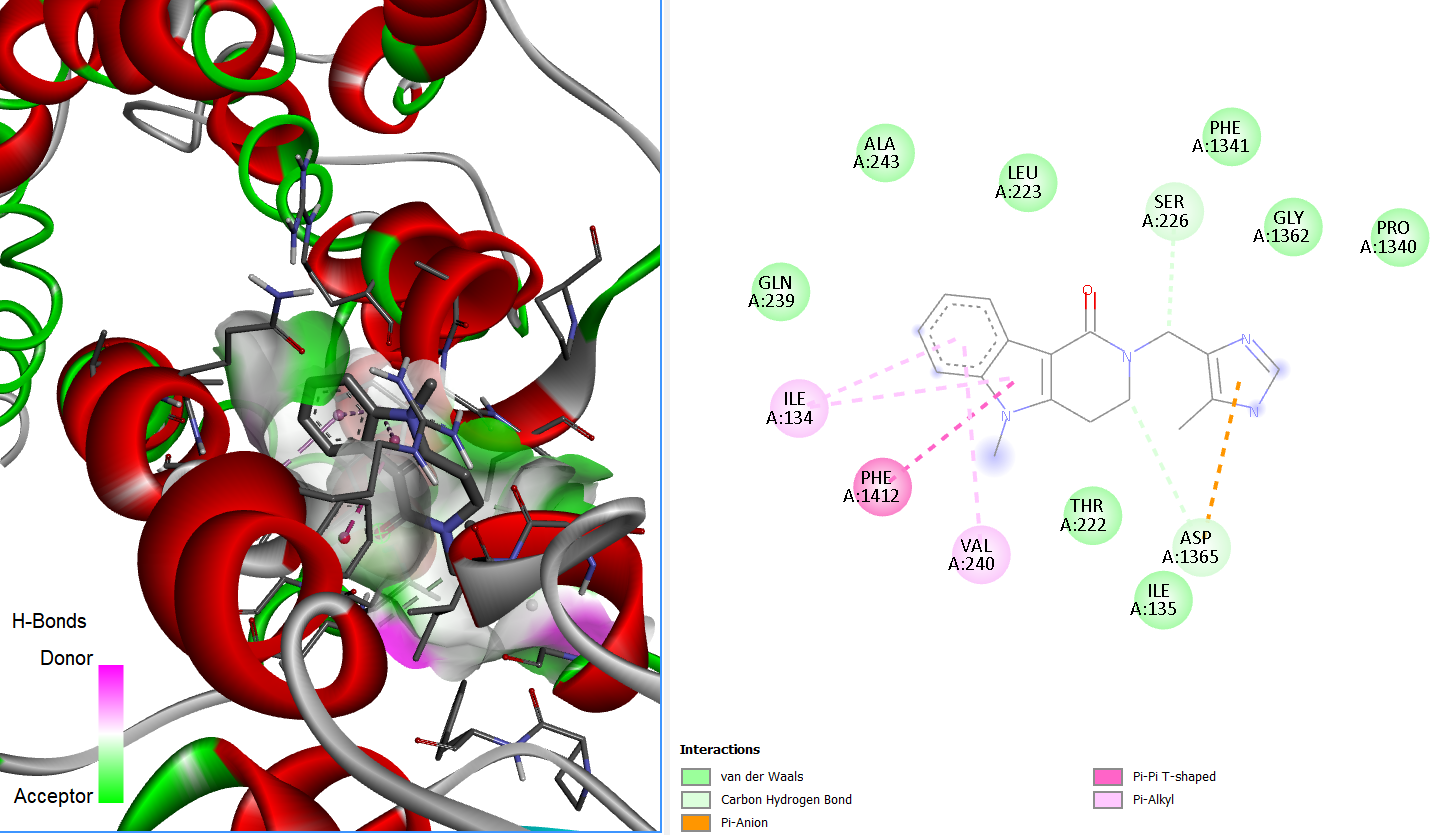


**Figure C.** NK1R-alosetron Docked complex 3D and 2D diagram.: Docked complexes of NK1R with ligands. In each complex, the violet and green color shades indicate hydrogen bonds. Each docked complex shows the 3D (left) and 2D (right) structure.


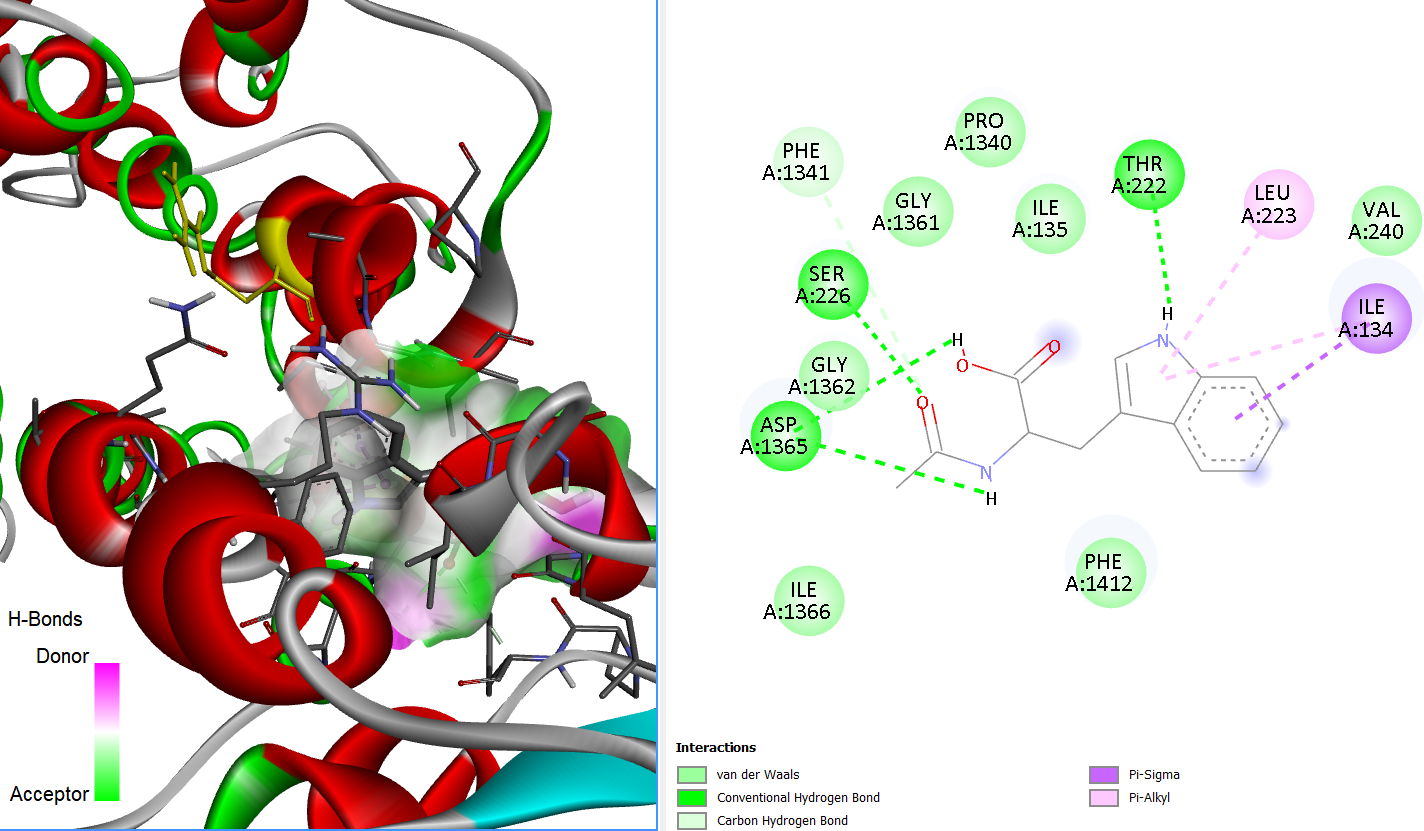


**Figure D.** NK1R-NAT tryptophan Docked complexes of NK1R with ligands. In each complex, the violet and green color shades indicate hydrogen bonds. Each docked complex shows the 3D (left) and 2D (right) structure.


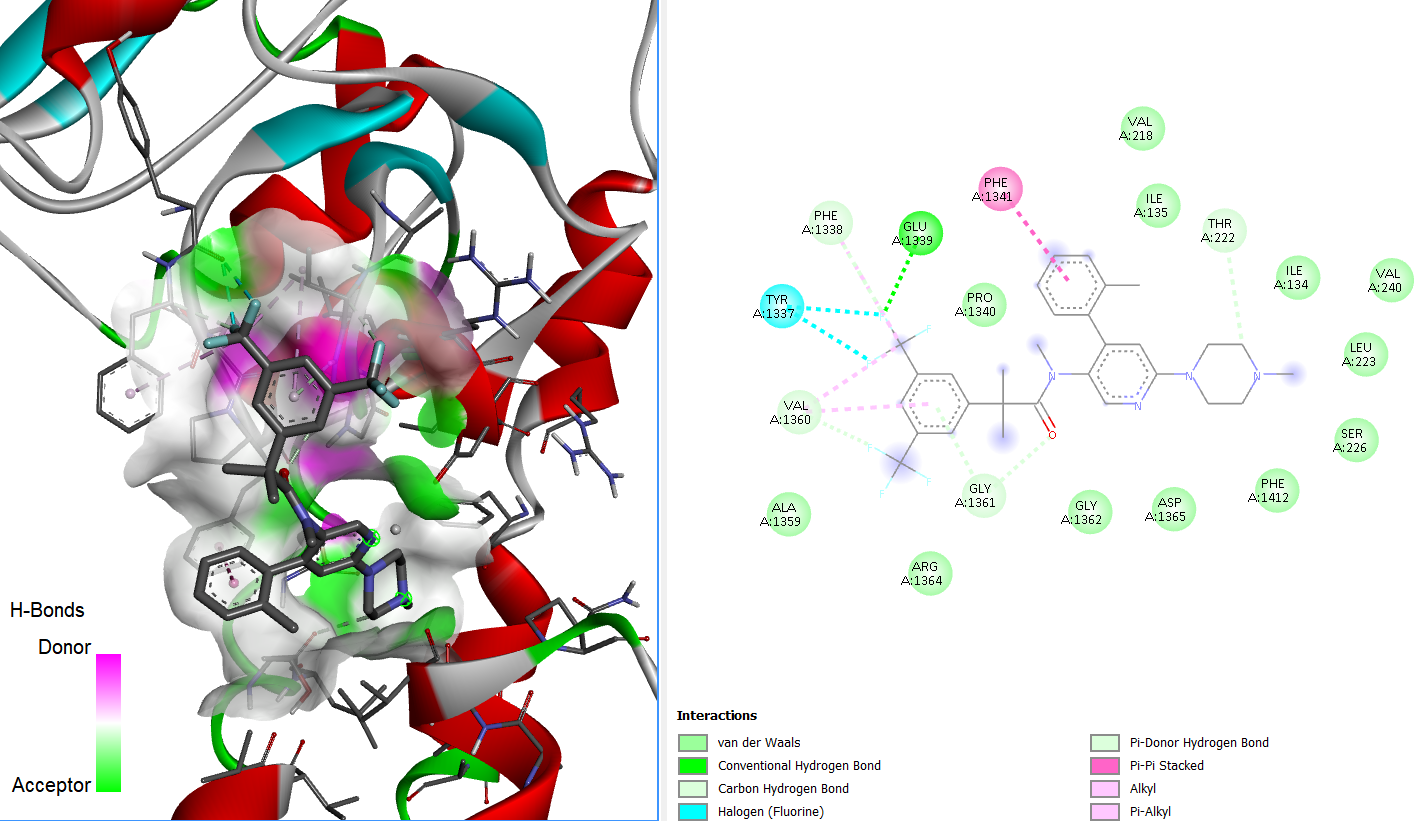


**Figure D.** NK1R-netupitant Docked complexes of NK1R with ligands. In each complex, the violet and green color shades indicate hydrogen bonds. Each docked complex shows the 3D (left) and 2D (right) structure.
