# Supplementary material for: Neuropeptide and cytokines expression in long COVID-19 related neuropsychological sequelae: insights into NK1R-mediated neuroinflammation and in silico therapeutic targeting
Source: Front Cell Neurosci. 2026 Mar 26;20:1763029. doi: 10.3389/fncel.2026.1763029 (PMC13061724; doi:10.3389/fncel.2026.1763029)
Supplement: Supplementary file 3 [file Data_Sheet_3.DOCX]

# Consent Form

**Long COVID-19 and Neurophyscological sequelae**

You are being asked to voluntarily participate in this study because you had been diagnosed with COVID-19 infection. We are inviting all eligible patients with COVID-19 infection to take part in this research. Your participation is completely voluntary and you have the right to refuse to be in this study. You can stop at any time after giving your consent. This decision will not affect in any way your current or future medical care or any other benefits to which you are entitled. The study investigator or sponsor may stop you from taking part in this study at any time if they decide it is in your best interest, or if you do not follow study instructions**.**

## Why is this study being done?

This study has done to evaluate some blood biomarkers that are related to post COVID-19 systemic complications and to identify potential therapeutic targets to reduce the risk of post COVID-19 complications. This will also enhance the quality of life in post COVID-19’s patients and will helpful to identify potential therapeutic targets which helps to reduced burden of post COVID-19 complications in Population

## How long will be in the study?

You will be in this study for only blood samples and will be contacted if required. However, if you decide to stop participating in the study, we encourage you to talk to the researcher and your doctor first.

## What are the risks of the study?

Because blood sample test will be obtain for research and will be done in schedule. After giving consent, you will not have to face additional risks .As in other circumstances; you might experience of pain, swelling, nerve injury, hematoma, infection, bruising and bleeding in puncture site. Some patients may also experience apprehension and anxiety due to their participation in study. If that is the case, we encourage you to discuss it with your doctor or researcher.

## Are there any benefits to taking part in the study?

Taking part in this study will not benefit you; however it may contribute to medical knowledge concerning the cause and careful monitoring of post COVID-19. Your participation may permit physicians in the future to better identify, monitor, treat and care for post COVID-19 patients.

## What about confidentiality?

You are also being asked to allow the study researchers to access and storage of identifying information from your medical record in our information data system. Your confidentiality will be maintained to the highest degree. The researchers, sponsor, Government agencies, Office for Human Research Protections (OHRP) and the Food and Drug Administration (FDA) may need to look at your medical and study records that contain yours protected health information. We will use a study number or code rather than your name on study records. Your name and other facts that might point to you will not appear when we present this study or publish its results.

## What are the costs?

There will be no added cost to you for participating in this study.

## What are my rights as a participant?

Taking part in this study is completely voluntary. You may choose not to take part or may leave the study at any time. Leaving the study will not result in any penalty or loss of benefits to which you are entitled. For more information, please contact: at 03446763687,

## Do I call if I have questions or problems?

For questions about the study, contact the researcher: For questions about your rights as a research participant, contact The University of Lahore. Institute of Molecular Biology and Biotechnology, near bobatian chowk, defense road Lahore.

## Voluntary Participation and Withdrawal:

Your participation is completely voluntary and you have the right to refuse to be in this study. You can stop at any time after giving your consent. This decision will not affect in any way your current or future medical care or any other benefits to which you are otherwise entitled.

By signing below you acknowledge that you have read (or a researcher/ doctor has read) and understand the material contained in this form and that you are willing to volunteer for this research.

Subject’s Printed Name: Subject’s signature:

Medical record number: Date Time

Subject’s Thumb impression Date Time

Witness (If Required)

Person Obtaining Consent

**Age: Sex: Address:**

**Thank you very much for your time, co-operation and support!**
