# Supplementary figures and images for: Neuropeptide and cytokines expression in long COVID-19 related neuropsychological sequelae: insights into NK1R-mediated neuroinflammation and in silico therapeutic targeting
Source: Front Cell Neurosci. 2026 Mar 26;20:1763029. doi: 10.3389/fncel.2026.1763029 (PMC13061724; doi:10.3389/fncel.2026.1763029)

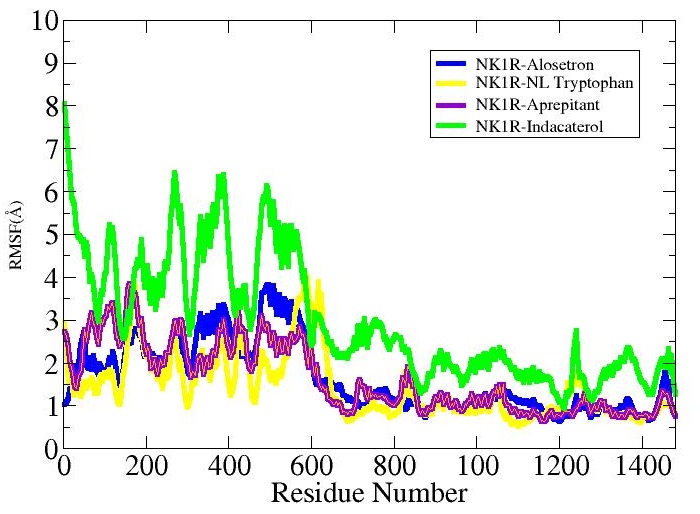

Supplement: Supplementary file 4 [file Image_1.JPEG]

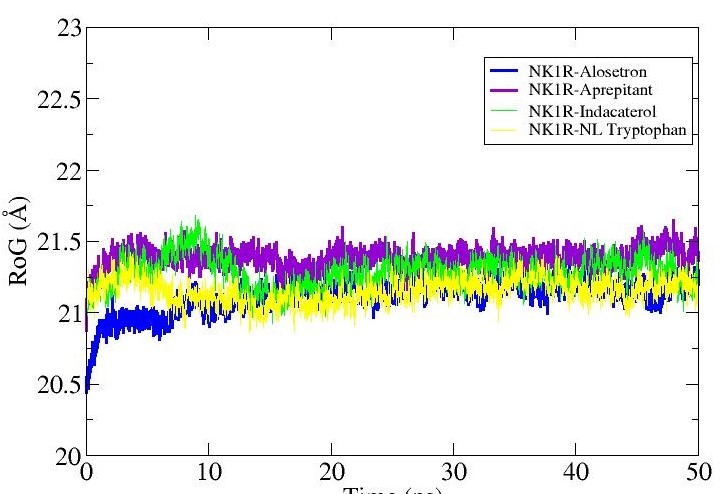

Supplement: Supplementary file 5 [file Image_2.JPEG]

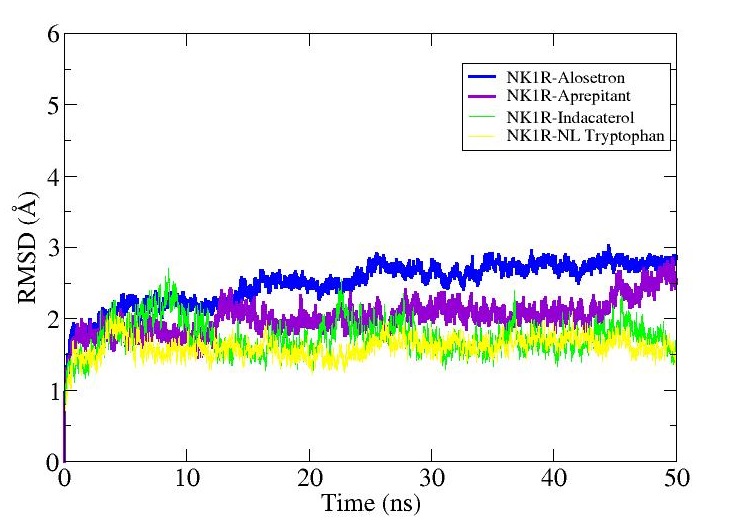

Supplement: Supplementary file 6 [file Image_3.JPEG]

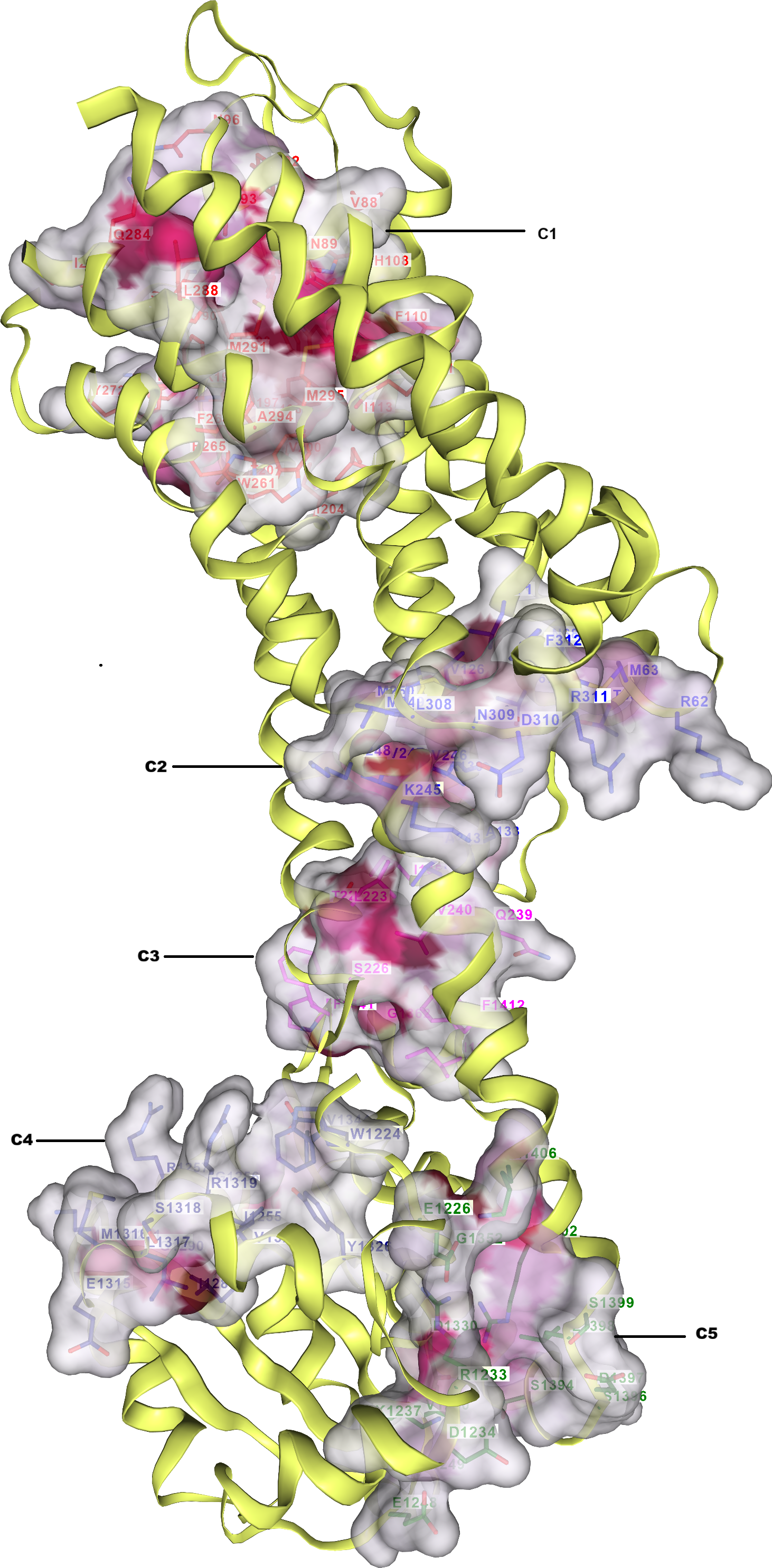

Supplement: Supplementary file 7 [file Image_4.PNG]
